# A systematic review and meta-analysis of the effects of probiotics on bone outcomes in rodent models

**DOI:** 10.1093/jbmr/zjae187

**Published:** 2024-11-15

**Authors:** Jenalyn L Yumol, William Gittings, Russell J de Souza, Wendy E Ward

**Affiliations:** Department of Kinesiology, Brock University, St. Catharines, ON L2S 3A1, Canada; Centre for Bone and Muscle Health, Brock University, St. Catharines, ON L2S 3A1, Canada; Department of Kinesiology, Brock University, St. Catharines, ON L2S 3A1, Canada; Centre for Bone and Muscle Health, Brock University, St. Catharines, ON L2S 3A1, Canada; Department of Health Research Methods, Evidence, and Impact, McMaster University, Hamilton, ON L8R 2K3, Canada; Department of Kinesiology, Brock University, St. Catharines, ON L2S 3A1, Canada; Centre for Bone and Muscle Health, Brock University, St. Catharines, ON L2S 3A1, Canada

**Keywords:** probiotics, bone QCT/μCT, DXA, preclinical studies, BMD, rodents

## Abstract

Emerging evidence demonstrates an opportunity for using probiotics to support bone health, but findings in humans are limited. This systematic review investigated if probiotic supplementation improves bone mineral density (BMD) and bone structure in rodent models compared to no supplementation. Studies (*n* = 71) examining the effect of oral consumption of any probiotic strain on BMD or bone structure in rodents were included. Meta-analyses were conducted separately by study model (intact, ovariectomized) and bone site (femur, tibia, spine) to determine the probiotic effect (standardized mean difference, SMD) on volumetric BMD (vBMD), bone volume fraction (BV/TV), and cortical thickness (Ct.Th). Reasons for heterogeneity were explored (probiotic genus, sex, type of rodent). In intact rodents, probiotics resulted in greater vBMD (SMD = 0.43, 95% CI [0.13, 0.74], *I*^2^ = 3%, *p* < 0.05) and higher BV/TV (SMD = 0.63, 95% CI [0.25, 1.02], *I*^2^ = 57%, *p* < 0.05) at the femur without changes in cortical bone structure. In ovariectomized models, probiotic supplementation resulted in greater vBMD (femur: SMD = 1.28, 95% CI [1.01, 1.55], *I*^2^ = 3%, *p* < 0.05; tibia: SMD = 1.29, 95% CI [0.52, 2.05], *I*^2^ = 67%, *p* < 0.05; and spine: SMD = 1.47, 95% CI [0.97, 1.97], *I*^2^ = 26%, *p* < 0.05) as well as higher BV/TV (femur: SMD = 1.16, 95% CI [0.80, 1.52], *I*^2^ = 56%, *p* < 0.05; tibia: SMD = 2.13, 95% CI [1.09, 3.17], *I*^2^ = 79%, *p* < 0.05; spine: SMD = 2.04, 95% CI [1.17, 2.90], *I*^2^ = 76%, *p* < 0.05) and Ct.Th at the tibia (SMD = 2.35; 95% CI [0.72, 3.97], *I*^2^ = 82%, *p* < .0.05) but not at the femur versus control. The syntheses support probiotics as a strategy to improve bone outcomes in rodent models.

## Graphical Abstract

**Graphical Abstract:**
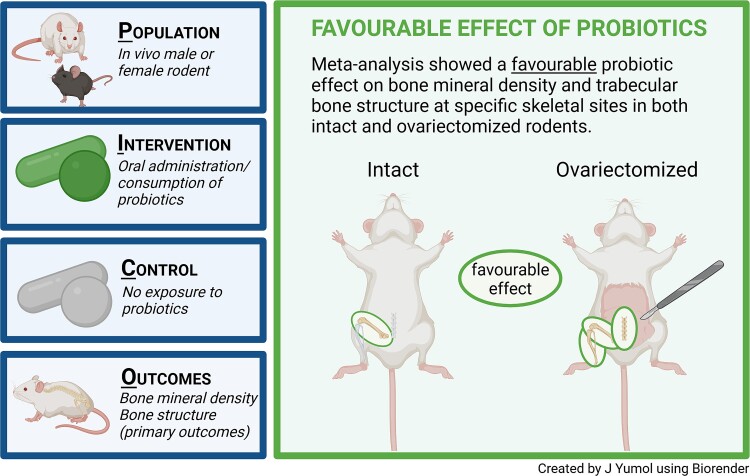

## Introduction

Probiotics are defined as “live microorganisms that, when administered in adequate amounts, confer a health benefit on the host”.^1^^,^^2^ Findings from studies using preclinical rodent models have suggested a role for probiotics as a regulator of bone cell metabolism via modulation of the gut microbiome—reducing permeability of the gut, maintaining the integrity of the mucosal barrier, reducing the production of proinflammatory cytokines, as well as increasing the production of bioactive metabolites, such as short-chain fatty acids, shown to stimulate bone formation or reduce bone resorption.^3–9^ However, few studies in humans have investigated the effect of probiotics on bone health, and the findings are equivocal.^10–13^ Such findings may be due to differences in bone health status (healthy, osteopenia), bacteria strains studied, bone site of analysis (hip, spine), as well as the study duration (6 or 12 months).

Rodent models are commonly utilized to explore the potential role of probiotics as bone-supporting factors because experimental designs can approximate various health conditions and life stages, as well as elucidate the sex- and probiotic strain-specific responses. The relationship between the gut microbiome and bone in rodents was previously demonstrated using a germ-free mouse model. At age 7 weeks, female germ-free mice had higher bone mineral density (BMD) at the tibia and femur, respectively, compared to conventionally raised mice.^14^ When germ-free mice were colonized with bacteria at young adulthood (age 3 weeks), BMD at the femur was normalized to levels similar to conventionally raised mice—tibia BMD was not reported.^14^ Similar findings were demonstrated in adult male mice (age 8 weeks) when germ-free mice were colonized with bacteria from conventional mice for 4 weeks.^15^ Others have demonstrated that the net effect observed in bone depended on the duration of the colonization period. For example, short-term bacterial colonization of 2 weeks resulted in increased bone resorption, demonstrating lower bone volume fraction (BV/TV) at the femur in colonized mice compared to germ-free mice, but increased bone formation was observed after 8 months of bacterial colonization resulting in a net increase of femur length in colonized male and female mice—the difference in BV/TV was not statistically significant.^16^ The interplay between the gut microbiome and bone metabolism presents an opportunity for using probiotics to modulate the gut microbiome as a potential strategy for maintaining bone health. The majority of studies measured BMD and bone structure as primary outcomes. The purpose of this systematic review and meta-analysis was to determine if probiotic supplementation improves these bone parameters in rodent models.

## Methods

The protocol was registered a priori on PROSPERO (https://www.crd.york.ac.uk/prospero/display_record.php?ID=CRD42021250351), and as per the PROSPERO revision note, studies that included periodontal disease were not included. This review was conducted in accordance with the Preferred Reporting Items for Systematic Reviews and Meta-Analyses (PRISMA) 2020 Checklist.^17^

### Search strategy

The databases searched were MEDLINE (Ovid), Embase (Ovid), CINAHL Complete (EBSCOhost), and Web of Science from inception to January 30, 2024, using Medical Subject Headings and keywords associated with probiotics, bone, and rodents. The full search strategy can be found in Table S1. Using Covidence (https://www.covidence.org/), titles and abstracts were screened independently by two reviewers (J.L.Y. and W.E.W. or W.G.). A third reviewer (W.E.W. or W.G.) was consulted to resolve any discrepancies, and inclusion or exclusion was decided by the majority consensus. Full text was screened by the primary reviewer (J.L.Y.) and uncertainties were reviewed among three reviewers (J.L.Y., W.E.W., and W.G.). ProQuest (Dissertations and Theses) and Google Scholar were used to search grey literature for the purpose of cross-referencing publications in peer-reviewed journals. Specifically, dissertations and theses were reviewed to help identify full-text publications in peer-reviewed journals that were referenced or first published as a dissertation or thesis. Dissertations and theses were not eligible for inclusion due to the lack of rigorous peer-review. The titles and abstracts, as well as the reference lists, of the first 80 search results identified using Google Scholar were reviewed to identify any missed articles that met the eligibility criteria of this systematic review. Unpublished literature was not eligible for inclusion.

#### Eligibility criteria

Included studies met the following criteria: in vivo male or female rodents at any life stage and disease model (excluding oral health conditions); oral administration/consumption of probiotic(s) of various strains with no restriction on dose or frequency of administration; non-exposed control group; intervention study with or without randomization; available data for BMD and/or bone structure analyses using any of the following methods: micro-computed tomography (μCT), dual energy X-ray absorptiometry (DXA), histology, dynamic bone histomorphometry, or specific bone cell staining. Secondary outcomes included measures of mechanical bone strength, serum markers of bone formation, bone resorption or inflammation, and bacteria from fecal samples. Studies were excluded if one or more of the following criteria were present: not an original study; not a full-text publication in English; models not using rodents; no oral administration/consumption of a probiotic intervention; none of the specified primary outcomes reported; studies with no comparator group(s). Justifications for exclusion of the full-text publications were recorded.

### Quality assessment and risk of bias

The quality of each study was examined using the Animal Research: Reporting of In Vivo Experiments (ARRIVE) guidelines 2.0.^18^ The guidelines consisted of 21 items describing the study planning, conduct of the study, and writing of the manuscript. The checklist was used to assess the adequacy of reported information within each study to support the evaluation of potential risk of bias. We previously adapted^19^ the risk of bias checklist based on the Systematic Review Center for Laboratory Animal Experimentation (SYRCLE’s) risk of bias tool.^20^ Using this, sequence generation (selection bias), baseline characteristics (selection bias), and incomplete outcome data (attrition bias) were assigned a “yes”—low risk of bias, “no”—high risk of bias, or “unclear” risk of bias prompted by a series of signaling questions (Table S2). The tool was revised for allocation concealment (selection bias), random housing (performance bias), blinding (performance bias), random outcome assessment (detection bias), blinding (detection bias), selective outcome reporting (reporting bias), and other sources of bias (other), whereby under the circumstance in which there was insufficient information to assign a “low” classification, then a “unclear” assessment was identified as it was assumed that these aspects were considered in some way (Table S2).

### Data extraction

A standardized Microsoft Excel form was used to extract the following data of included studies: study identification, study design, animal model, intervention, primary outcomes (BMD, trabecular and cortical bone structure outcomes, adverse events), and secondary outcomes (bone strength, serum biomarkers of bone formation, resorption or inflammation, gut microbiota composition). Authors were contacted via email to request for missing or unclear data. If there was no response within 7 days, and it was possible from the presented figures, two reviewers (J.L.Y., P.W.F.) independently extracted the data from the figure(s) using WebPlot Digitizer (https://apps.automeris.io/wpd/).

### Data synthesis

Using Review Manager (RevMan) Version 5.4.1 software,^21^ the effect of the probiotic intervention on BMD, trabecular bone structure outcomes, and cortical bone structure outcomes were reported as the standardized mean difference (SMD) between the probiotic group and control group for each study. Quantitative syntheses were conducted separately by study model (intact, ovariectomized [OVX]) and bone site of analysis (femur, tibia, spine), provided there were at least two articles. SMD were pooled using DerSimonian and Laird random-effects meta-analysis, and the corresponding 95% confidence intervals (CI) were generated around the pooled results for volumetric BMD (vBMD), trabecular bone structure outcomes (BV/TV; trabecular thickness, Tb.Th; trabecular number, Tb.N; trabecular separation, Tb.Sp), and cortical bone structure outcomes (cortical thickness, Ct.Th; cortical area, Ct.Ar). All animals were only counted once in a single forest plot. Where investigators re-used a single control group to produce multiple comparisons, we corrected the standard errors by splitting the sample size into the number of groups compared. If the total sample size for the control group was an uneven number, the split sample size number was rounded down. Reasons for large heterogeneity (*I*^2^ *>* 50) were explored using meta-regression implemented in Stata/SE 16.1 software,^22^ including sex (females, males), probiotic genus (*Lactobacillus*, *Bifidobacterium*, mix of *Lactobacillus*, and *Bifidobactium*, other genus), and type of rodent (mice, rats). Publication bias was estimated using Egger’s regression and a visual inspection of funnel plots—symmetrical sides were regarded as no publication bias present. If necessary, a trim-and-fill analysis was conducted when at least 10 studies were available to account for publication bias.

## Results

**Figure 1:**
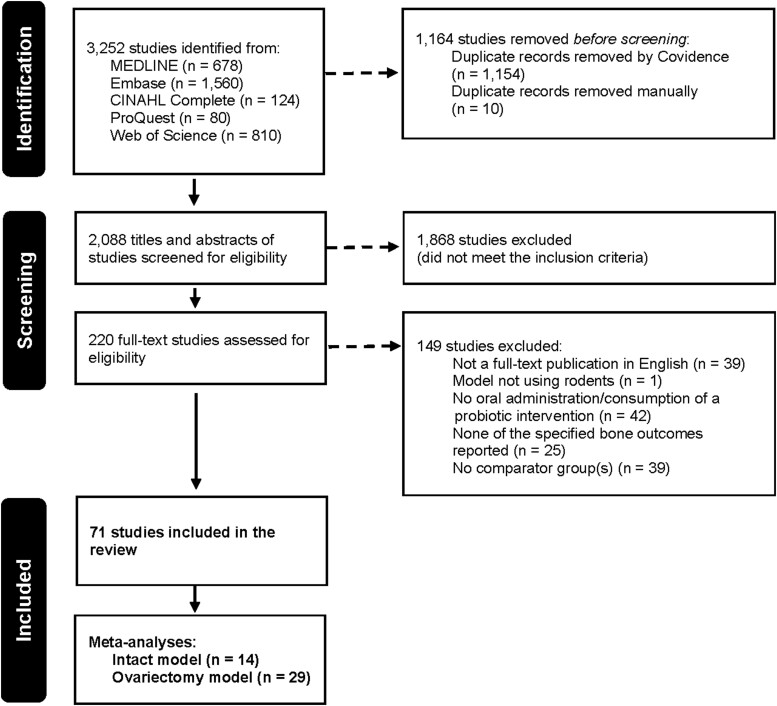
PRISMA flow diagram.

A total of 3252 articles were identified using MEDLINE (*n* = 678) and Embase (*n* = 1560), CINAHL Complete (*n* = 124), ProQuest Dissertations and Theses (*n* = 80), and Web of Science (*n* = 810). No additional articles were found after searching grey literature and reference lists of relevant publications. After the removal of 1164 duplicates, 2088 studies were included for title and abstract screening. From the list, 220 studies passed through to full-text review and were assessed for eligibility, at which stage 149 articles were excluded for one or more of the following reasons: not a full-text publication in English, not a rodent model, no oral administration or consumption of a probiotic intervention, none of the specified primary outcomes reported, or studies with no comparator group(s). Overall, this systematic review consisted of data synthesis from 71 included studies (Figure 1).

### Characteristics of included studies

Of the number of studies included in our analyses, 63% were conducted in mice (*n* = 45 studies) and 61% were in females (*n* = 43 studies), compared to rats and males, respectively. The effect of probiotics on bone outcomes was most commonly examined using an intact model [*n*_mice_ = 93 females^3^^,^^23–26^ and 182 males,^27–35^  *n*_rats_ = 10 females^8^ and 16 males^36^; Table S3] or an OVX model [*n*_mice_ = 402 females,^3^^,^^6^^,^^9^^,^^23–25^^,^^37–48^  *n*_rats_ = 325 females,^8^^,^^27^^,^^49^^,^^50^^,51–60^; Table S4]. Other study models included co-intervention with a high-fat diet [*n*_mice_ = 12 females^61^ and 40 males,^32^^,62^], diabetes [*n*_mice_ = 14 males^29^], arthritis [*n*_mice_ = 61 males,^49^^,63^, *n*_rats_ = 34 males^64,65^ and 12 undefined sex^66^], fracture healing [*n*_mice_ = 20 females^67^ and 105 males^68,69^], glucocorticoid-induced osteoporosis [*n*_mice_ = 48 males,^4^^,70^, *n*_rats_ = 15 females^71^ and 32 males^72^], tenofovir disoproxil fumarate-induced bone loss [*n*_mice_ = 20 males^73^], retinoic-induced secondary osteoporosis [*n*_rats_ = 32 females^74,75^], CoCrMo particle-induced osteolysis [*n*_mice_ = 10 sex not defined^76^], ulcerative colitis [*n*_mice_ = 56 males^77,78^], dorsal incision surgery [*n*_mice_ = 14 females^26^], depression [*n*_rats_ = 16 males^79^], cigarette smoking inhalation [*n*_rats_ = 16 males^36^], chronic mild stress [*n*_rats_ = 24 males^80^], bone cancer pain [*n*_rats_ = 12 females^81^], co-intervention with antibiotics [*n*_mice_ = 36 males^5^], or genetic modification [*n*_mice_ = 96 females^7^], as well as an orchidectomy model [*n*_mice_ = 28 males^35^] (Table S5).

From 35 bacterial species, a total of 61 identified strains and 18 unidentified strains were examined over an average intervention duration of 7.95 ± 5.24 weeks [median (min-max) = 6(2 − 26) weeks]. The probiotic intervention was provided orally via gavage, diet, or within drinking water, either independently or in combination with other strains of the same or different species. The majority of bacteria species studied were from the *Lactobacillus* (37%) or *Bifidobacterium* genera (26%); other genera studied included *Bacillus*, *Bacteroides*, *Enterococcus*, *Streptococcus*, *Akkermansia*, *Faecalibacterium*, *Clostridium*, *Prevotella*, and *Propionibacterium* (Table S6).

### Quality of evidence

**Figure 2:**
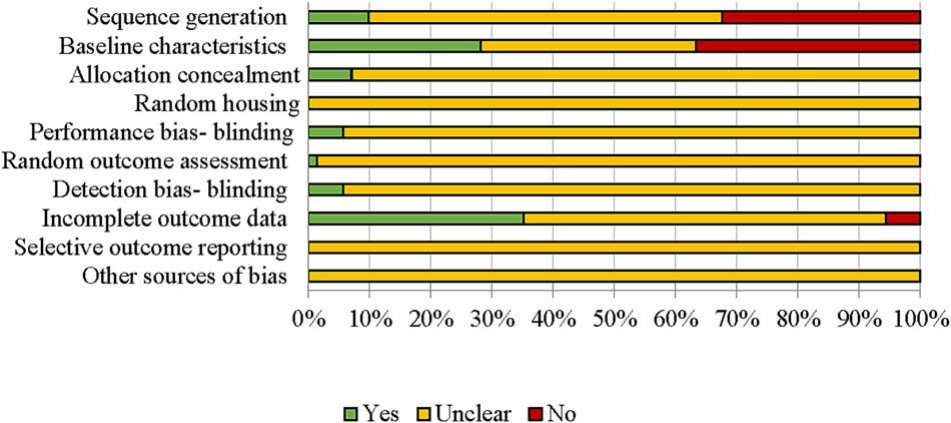
Risk of bias assessment results using SYRCLE’s tool.

Findings from the quality assessment using the ARRIVE guidelines (Table S7) suggested that suboptimal reporting within the studies contributed to the large proportion of unclear classifications in SYRCLE’s risk of bias assessment (Figure 2).

#### Selection bias

A description of the random component in the allocation sequence generation was reported in 9.86% of the studies (*n* = 7); however, two of these studies provided further details of the baseline characteristics to support the determination of whether the groups were comparable at baseline. Although one study reported controlling groups for body weight at baseline, the lack of difference in initial body weights among intervention groups in 28.17% of the studies (*n* = 20) implied consideration for weight-matched groups. Findings from the ARRIVE assessment suggested inconsistent reporting of study design [“yes”: (a) groups compared = 100%; (b) experimental unit = 35.21%], along with methods for randomization [“yes”: (a) methods = 16.90%; (b) confounders = 30.99%]. Details for allocation concealment were provided for 7.04% of the studies (*n* = 5), and the limited blinding reported was demonstrated using the ARRIVE assessment, in which only 12.68% of the studies described methodology for blinding at any stage of the study.

#### Performance bias

Random housing of the rodents (100%) and blinding of the investigators (94.37%) were unclear due to the lack of reported experimental unit and randomization methodology and blinding, respectively (described above). One study reported switching bedding and mice between cages during the acclimatization period to limit cage effects on the microbiome.^67^

#### Detection bias

One study provided details regarding random outcome assessment. Like performance bias, 94.37% of studies (*n* = 67) did not report measures used to blind the outcome assessor(s).

#### Attrition bias

A total of 35.21% of the studies (*n* = 25) had no exclusions or clearly explained the sample size between the methods and results, either in the inclusions and exclusions, statistical analyses, or results section.

#### Reporting bias

A priori registration of preclinical trials is currently not typical nor a mandatory requirement, making it difficult to assess reporting bias. No study indicated protocol registration, resulting in unclear selective outcome reporting for all studies (*n* = 71).

### Primary outcomes and meta-analyses

Results were reported separately by study model: intact model, OVX, and other. BMD and bone structure were examined using μCT or DXA and trabecular and cortical bone structure outcomes were quantified using μCT or histologic methodology at the femur, tibia, or spine. A meta-analysis was conducted for the following measures obtained using μCT: vBMD, BV/TV, Tb.Th, Tb.Sp, Tb.N, Ct.Th, and Ct.Ar.

#### Intact model

This classification included male and female rodents with intact gonads and no additional genetic modification.^3^^,^^7^^,^^8^^,^^23–26^^,^^28–32^^,^^34–36^^,82^

##### Bone mineral density

In male mice approximately 14 weeks of age, *Lactobacillus reuteri* ATCC PTA 6475 provided at least three times per week for 4 weeks at 1 × 10^9^ CFU/mL resulted in greater BMD at the femur^28^^,^^31^ and spine.^28^ Conversely, the bone promoting effect of *L. reuteri* ATCC PTA 6475 was not observed by Zhang et al.,^29^ in a similar rodent model. There was no effect on BMD at the femur following a daily consumption (1 × 10^9^ CFU/mL) of *Lactiplantibacillus* (formerly *Lactobacillus*) *plantarum* TWK10 in older male mice (age 17 or 82-95 weeks) for 8 weeks^(82)^, *Lacticaseibacillus* (formerly *Lactobacillus casei*) *paracasei* DSM 13434 and *Lactiplantibacillus plantarum* DSM 15312 mixture in male mice (age 10 weeks) for 6 weeks,^35^ or *Bifidobacterium pseudocatenulatum* in younger male mice (age 7-9 weeks) for 14 weeks.^32^ There was also a lack of probiotic effect on BMD at the femur in female mice (age 8 weeks) provided *Lactobacillus paracasei* or *Lactobacillus* mixture for 6 weeks at 1 × 10^8^ CFU.^24^ However, lower BMD was observed in female mice aged 12 weeks following a 4-week daily administration of 2 × 10^8^ CFU pasteurized *Akkermansia muciniphila* (p*AKK*).^23^

##### Trabecular and cortical bone structure

Effects on bone structure outcomes were inconsistent. Greater BV/TV, Tb.Th, Tb.N and lower Tb.Sp at the femur and spine were reported in male mice receiving *L. reuteri* ATCC PTA 6475 [0.3 mL of 1 × 10^9^ CFU/mL three times per week^28^]. Others demonstrated little to no effect on bone structure.^29^^,^^31^^,82^ One study investigated heat-activated and inactive *Lactobacillus acidophilus* ATCC 4356 [400 μL daily], and while the inactive form demonstrated no effect on trabecular bone structure of the femur, male mice provided the heat-activated form had greater BV/TV and Tb.N along with lower Tb.Sp compared to the control group.^34^ An increase in BV/TV at the femur, but not the spine, was shown following administration of 1 × 10^9^ CFU/mL *Lactobacillus rhamnosus* GG (LGG) twice a week for 4 weeks.^3^ It was later demonstrated that daily administration resulted in greater BV/TV at both the femur and spine.^7^ In contrast, lower Tb.Th and Ct.Ar at the spine were reported following 4-week p*AKK* treatment [2 × 10^8^ CFU/mL daily^23^]. All other studies reported no significant effect on bone structure at the femur, tibia, or spine.^3^^,^^8^^,^^24^^,^^25^^,^^30^^,^^32^^,^^33^^,^^35^ Few studies investigated bone outcomes using dynamic histomorphometry. Greater bone formation rate (BFR) and/or mineral apposition rate (MAR) were observed in studies that reported improved vBMD or bone structure using μCT,^7^^,^^28^ and null findings were reported in studies with no effect on these outcomes of bone health.^29^^,^^31^

##### Meta-analyses

**Figure 3:**
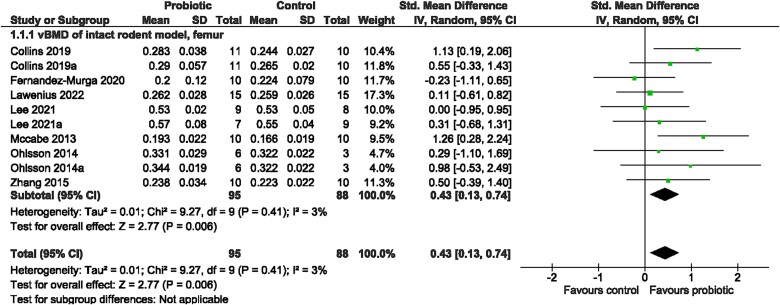
Intact rodent model. The effect of probiotic intervention on vBMD of the femur. Abbreviations: CI, confidence interval; df, degrees of freedom; IV, weighted mean difference; SD, standard deviation.

**Figure 4:**
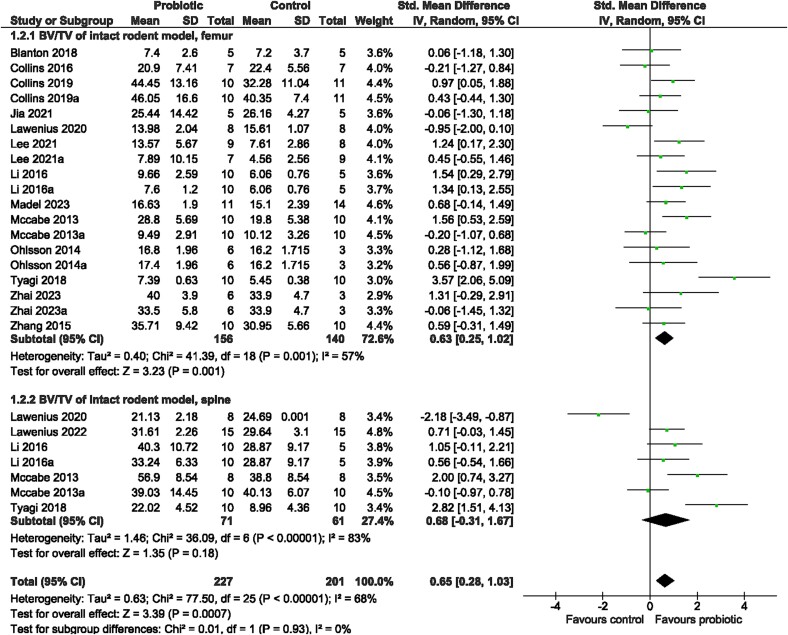
Intact rodent model. The effect of probiotic intervention on BV/TV of the femur and spine. Abbreviations: CI, confidence interval; df, degrees of freedom; IV, weighted mean difference; SD, standard deviation.

**Figure 5:**
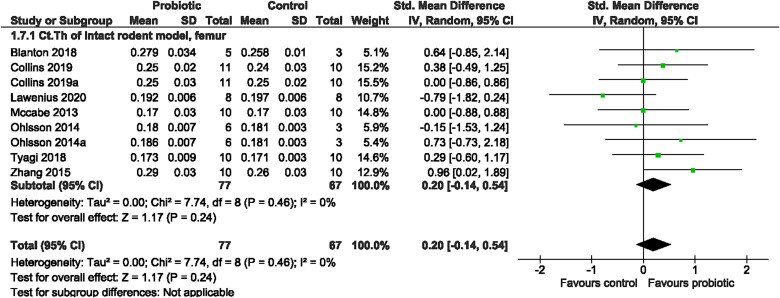
Intact rodent model. The effect of probiotic intervention on Ct.Th of the femur. Abbreviations: CI, confidence interval; df, degrees of freedom; IV, weighted mean difference; SD, standard deviation.

Probiotic supplementation, in general, resulted in significantly greater vBMD at the femur (SMD = 0.43, 95% CI [0.13, 0.74], *I*^2^ = 3%, *p* = 0.006; Figure 3), which was consistent with greater BV/TV (SMD = 0.63, 95% CI [0.25, 1.02], *I*^2^ = 57%, *p* = 0.001, Figure 4) and Tb.Th (SMD = 0.66, 95% CI [0.25, 1.07], *I*^2^ = 55%, *p* = 0.001; Figure S1) as well as lower Tb.Sp (SMD = −0.37, 95% CI [−0.67, −0.07], *I*^2^ = 26%, *p* = 0.02; Figure S2), compared to the control. There was no effect on BV/TV at the spine (SMD = 0.68, 95% CI [−0.31, 1.67], *I*^2^ = 83%, *p* = 0.18; Figure 4), Tb.N at the femur (SMD = 0.42, 95% CI [−0.01, 0.84], *I*^2^ = 61%, *p* = 0.05; Figure S3) or for cortical bone outcomes at the femur, including Ct.Th (SMD = 0.20, 95% CI [−0.14, 0.54], *I*^2^ = 0%, *p* = 0.24; Figure 5) and Ct.Ar (SMD = 0.13, 95% CI [−0.16, 0.41], *I*^2^ = 0%, *p* = 0.38; Figure S4). Further investigation was done for outcomes with large heterogeneity, in which probiotic genus had a significant effect (*p* = 0.016) on BV/TV, but not sex (*p* = 0.853), site (*p* = 0.986), or rodent type (*p* = 0.818). Sex, probiotic genus, or rodent type had no effect (*p* > 0.05) on Tb.Th or Tb.N at the femur. Visual inspection of the funnel plots and findings from the Egger regression suggested no evidence of publication bias for vBMD, BV/TV, Tb.Sp, Tb.Th, Ct.Th, or Ct.Ar (Figure S5A-C and E-G). Findings from the Egger regression suggested publication bias was present for Tb.N, and a trim-and-fill analysis was performed, resulting in one imputed study (Figure S5D).

#### Ovariectomy model

OVX is a well-established rodent model used to mimic the rapid decline of estrogen in women following menopause.^83^ The depletion of estrogen induces unfavorable bone changes compared to a sham-operated control group.^3^^,^^6^^,^^8^^,^^9^^,^^23–25^^,^^27^^,^^37–50^^,51–58,60,84–87^

##### Bone mineral density

Probiotic supplementation mitigated the deleterious effects of the OVX surgery on bone outcomes. The probiotic effect on BMD was irrespective of intervention duration (mice = 4-9 weeks; rats = 4-16 weeks), but findings suggest the effects to be strain specific. Greater BMD at the femur, tibia, and spine were demonstrated following daily administration of *Bacteroides vulgatus* ATCC 8482,^42^  *Prevotella histicola*,^44^  *Rothia*^48^ for 8 weeks; *Bifidobacterium longum* UBBL-64M1395,^45^  *Lactobacillus brevis* AR281,^86^  *Lactobacillus plantarum* AR495^9^ for 7 weeks; *Bacillus clausii*,^37^  *L. acidophilus* ATCC 4356,^38^ or *L. rhamnosus* UBLR-58 MTCC 5402 for 6 weeks at 1 × 10^9^ CFU/mL.^39^ Conversely, there was no effect on femur BMD using *L. plantarum* AR237,^9^  *L. paracasei*, or *Lactobacillus* mixture.^41^ Although, other probiotics may exert an effect at a shorter intervention duration, in which *L. reuteri* ATCC PTA 6475,^6^  *B. longum* 35624,^43^  *L. paracasei* GKS6,^42^ as well as, *L. paracasei* GML-678 or GML-653,^84^ demonstrated greater BMD at 4 weeks at the femur, spine, and tibia, respectively, when compared to the OVX control group. Observed effects in rat studies were similar to those reported in mice. When compared to the control, greater BMD was reported at the femur when *B. longum* ATCC 15707 or *Lactobacillus helveticus* ATCC 27558 was provided daily for 16 weeks at 1 × 10^8-9^ CFU/mL in rats age 14 weeks.^50^^,51^ This was supported by greater OBS/BS, OS/BS and OV/BV along with lower OcS/BS and ES/BS with *B. longum* ATCC 15707 but not *L. helveticus* ATCC 27558. Older rats (age 26 weeks) had a similar positive response to daily administration of *L. rhamnosus* HN001 (1 × 10^9^ CFU) at 12 weeks but not 4 weeks.^52^ Greater whole-body BMD was also observed at 16 weeks following oral administration of *Lactobacillus interstinalis* KCTC 5052 or KCCM11812P three times per week,^27^ but no effect was seen at the tibia using *L. plantarum* CJNU3003 daily intervention^87^ nor at the femur of younger rats aged 6 weeks provided *Lactobacillus gasseri* daily for 8 weeks.^56^ Interestingly, inactive *Propionibactrium freudenreichii* MJ2, but not the active form, demonstrated a positive effect on BMD at the femur following 17 weeks of daily intervention at either a low dose of 1 × 10^7^ CFU/mL or a high dose of 1 × 10^8^ CFU/mL.^49^ This same study also provided a proof of the principle for bone promoting effects of their positive control, *L. plantarum.*^49^ Tsai et al. investigated probiotic dosages and suggested a low dose (2.07 × 10^8^) of *L. plantarum* GMNL-662 had no effect at the femur but higher doses (4.13 × 10^8^ and 8.27 × 10^8^) resulted in greater BMD at the femur in rats age 12 weeks.^60^ Using a shorter intervention duration of 6 weeks, greater BMD at the femur was reported following daily administration of LGG ATCC 7469.^55^ Daily administration of *L. casei*, *Bacillus coagulans*, *Bifidobacterium*, *L. reuteri*, *L. rhamnosus*, or *Lactobacillus* and/or *Bifidobacterium* mixtures was also studied, but inconsistent results were reported at 4 weeks for each probiotic treatment on BMD at the tibia, spine or whole-body.^53,54,57,58^

##### Trabecular and cortical bone structure

Unlike findings using an intact model, favorable bone changes were more consistently shown in OVX rodents when provided a probiotic treatment. Greater BV/TV, Tb.Th, Tb. N, Ct.Ar, Ct.Th, connectivity density (Conn.Dn), total area (T.Ar), total perimeter of the bone (T.Pm), and/or periosteal perimeter (Ps.Pm) along with lower Tb.Sp were observed at the femur, tibia and spine with *B. clausii* [6 weeks; 1 × 10^9^ CFU/mL daily^37^], *L. acidophilus* ATCC 4356 [6 weeks; 1 × 10^9^ CFU/mL daily^38^] or GDMCC1.412 [6 weeks, 1 × 10^8-9^ CFU/mL daily^46^], LGG ATCC7469 [6 weeks; 1 × 10^9^ CFU/mL daily^55^], *L. rhamnosus* UBLR-58 MTCC 5402 [6 weeks; 1 × 10^9^ CFU/mL daily^39^], *Lactobacillus salivarius* LI01 [6 weeks; 3 × 10^9^ CFU/mL daily^85^], *L. paracasei* GMNL-653 (inactive) or GMNL-678 (active) [4 weeks; 1 × 10^10^ CFU/mL daily^84^], *L. plantarum* GMNL-662 [14 weeks; 2.07, 4.13 or 8.27 × 10^8^ CFU/kg daily^60^], AR237 or AR495 [7 weeks; 1 × 10^9^ CFU/mL daily^9^], *B. longum* UBBL-64M1395 [7 weeks; 1 × 10^9^ CFU/mL daily^45^], *B. vulgatus* ATCC 8482 [8-9 weeks; 5 × 10^9^ CFU/mL every 2 d^42^], inactive *Propionibacterium freudenreichii* MJ2 [17 weeks; 1 × 10^7^ or 10^8^ CFU/mL daily^49^], Lifespace probiotic [containing a mixture of *L. rhamnosus* HN001, *Bifidobacterium lactis* BI-04, *Bifidobacterium animalis* HN019, *Lactobacillus fermentum* SBS-1, *L. reuteri* 1e1, *B. longum* BB536, *Bifidobacterium breve* M16-V, *Bifidobacterium infantis* Bi-26, *L. paracasei* Lpc-37; 7 weeks; 1 × 10^7^ CFU/mL daily^8^], *Saccharomyces boulardii* [6 weeks; 3 g/kg body weight three times per week^25^], and *Rothia* [8 weeks; 1 × 10^8^ CFU/mL daily^48^]. *L. reuteri* ATCC PTA 6475 (1 × 10^9^ CFU/mL) administration three times per week for 4 weeks resulted in a favourable trabecular bone response at the femur, but no effect was seen at the spine—supported by no effect on BFR.^6^ No differences in cortical bone outcomes in the femur were reported for study groups.^6^ Bone site differences were also reported with greater BV/TV at the femur but not spine with LGG or VSL#3 [containing a mixture of *B. breve*, *B. longum*, *B. infantis*, *L. acidophilus*, *L. plantarum*, *L. paracasei*, *Lactobacillus bulgaricus*, *Streptococcus thermophilus*; 4 weeks; 1 × 10^9^ CFU twice per week^3^]. Little to no effect on bone structure was demonstrated following administration of *L. plantarum*, strain undefined [4 weeks; 2 × 10^11^ CFU/g daily^40^] or CJNU 3003 [16 weeks; 1 × 10^8^ CFU/kg daily^87^], *L. paracasei*, strain undefined [4 weeks; 2 × 10^11^ CFU/g daily^40^], DSM 13434 alone or with DSWM 15312 and DSM 15313 [5.5-6 weeks; 1 × 10^8^ CFU/mL daily^24^^,^^41^], *L. reuteri* GDMCC1.614 alone or combined with *L. acidophilus* GDMCC1.412 [6 weeks; 1 × 10^8-9^ CFU/mL daily], *L. gasseri* [8 weeks; 1 × 10^9^ CFU daily^56^], *L. rhamnosus* alone or combined with azithromycin (AZM) antibiotic [4 weeks; 3 × 10^8^ CFU/kg daily^57^], *L. helveticus* ATCC 27558 or *B. longum* ATCC 15707 [16 weeks; 1 × 10^8-9^ CFU/mL daily,^50^^,51^], *P. histicola* [8 weeks; 1 × 10^8^ CFU/mL daily^44^ or 12 weeks; 1 × 10^9^ CFU every other day^47^], or p*AKK* [4 weeks; 2 × 10^8^ CFU daily^23^].

##### Meta-analysis

**Figure 6:**
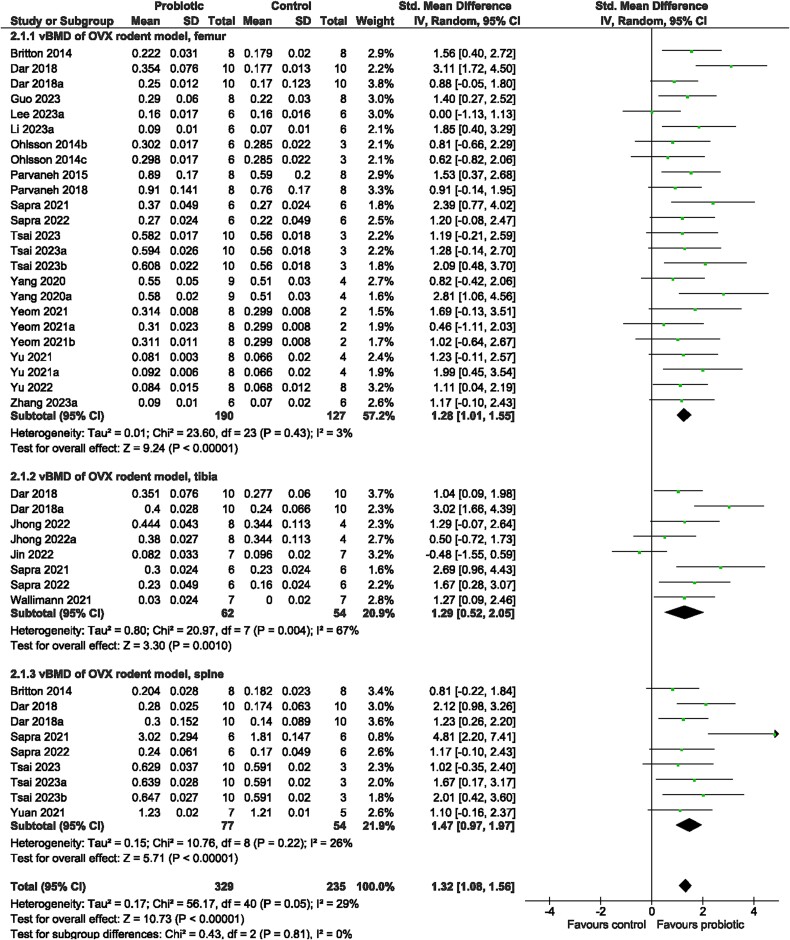
Ovariectomized (OVX) rodent model. The effect of probiotic intervention on vBMD of the femur, tibia, and spine. Abbreviations: CI, confidence interval; df, degrees of freedom; IV, weighted mean difference; SD, standard deviation.

**Figure 7:**
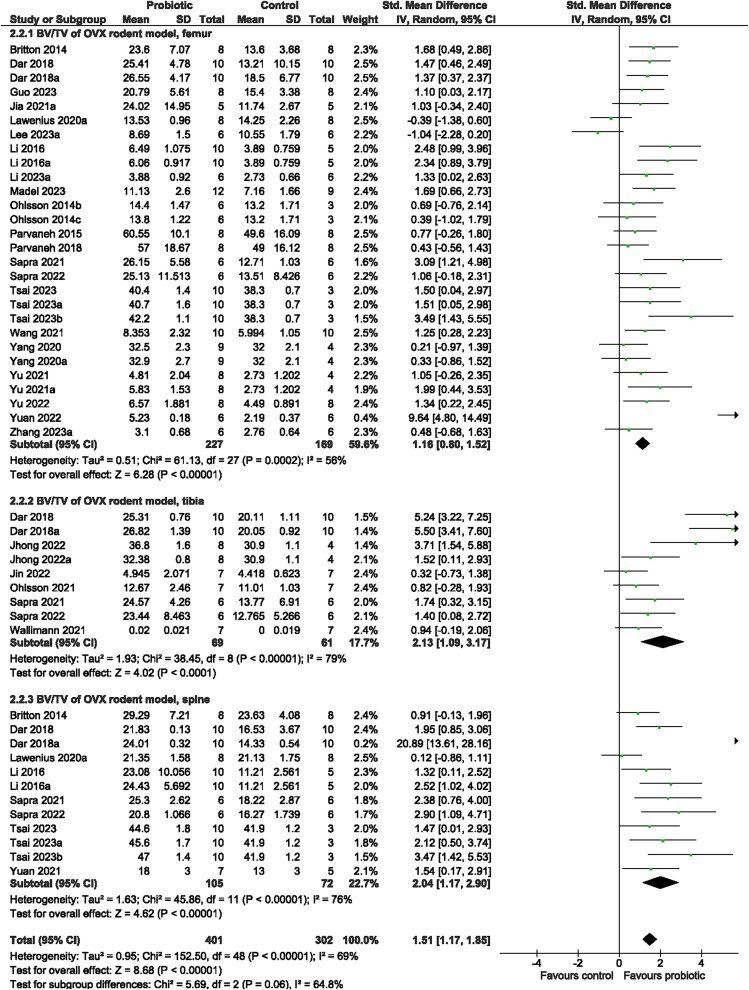
Ovariectomized (OVX) rodent model. The effect of probiotic intervention on BV/TV of the femur, tibia, and spine. Abbreviations: CI, confidence interval; df, degrees of freedom; IV, weighted mean difference; SD, standard deviation.

**Figure 8:**
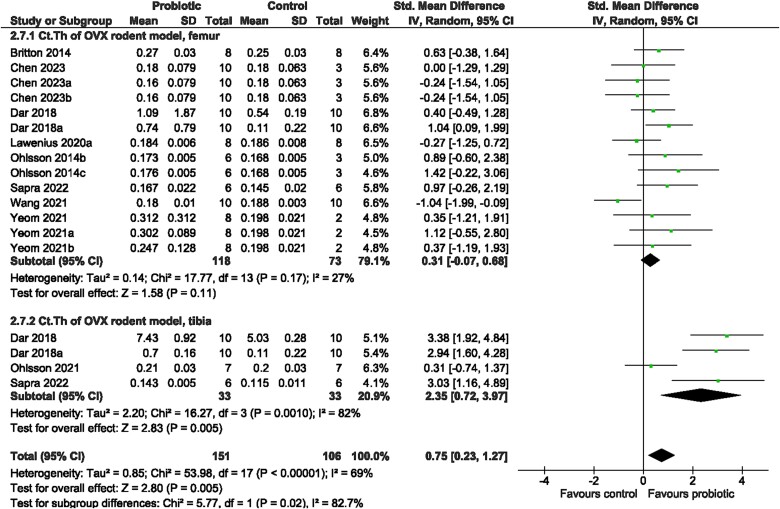
Ovariectomized (OVX) rodent model. The effect of probiotic intervention on Ct.Th of the femur and tibia. Abbreviations: CI, confidence interval; df, degrees of freedom; IV, weighted mean difference; SD, standard deviation.

Irrespective of bone site examined (test for subgroup differences [femur, tibia, spine]: *p* > 0.05), rodents receiving probiotic supplementation demonstrated a benefit on trabecular bone structure compared to control animals. There was significantly greater vBMD (SMD = 1.32, 95% CI [1.08, 1.56], *I*^2^ = 29%, *p* < 0.00001; Figure 6), BV/TV (SMD = 1.51, 95% CI [1.170, 1.85], *I*^2^ = 69%, *p* < 0.00001; Figure 7), Tb.Th (SMD = 0.96, 95% CI [0.67, 1.26], *I*^2^ = 56%, *p* < 0.00001; Figure S6), and Tb.N (SMD = 1.23, 95% CI [0.86, 1.59], *I*^2^ = 68%, *p* < 0.00001; Figure S7) and lower Tb.Sp (SMD = −1.20, 95% CI [−1.55, −0.85], *I*^2^ = 68%, *p* < 0.00001; Figure S8) with probiotic supplementation. The probiotic effect on cortical bone outcomes was observed at the tibia but not femur—greater Ct. Th (tibia: SMD = 2.35, 95% CI [0.72, 3.97], *I*^2^ = 82%, *p* = 0.005; femur: SMD = 0.31, 95% CI [−0.07, 0.68], *I*^2^ = 27%, *p* = 0.11; Figure 8) and Ct. Ar (tibia: SMD = 0.84, 95% CI [0.28, 1.41], *I*^2^ = 0%, *p* = 0.004; femur: SMD = 1.03, 95% CI [−0.03, 2.09], *I*^2^ = 82%, *p* = 0.06; Figure S9)—in rodents provided the probiotic supplementation. Probiotic genus or rodent type were not reasons for the large heterogeneity (*p* > 0.05) among studies for vBMD, BV/TV, Tb.Th, Tb.N, Tb.Sp, or Ct.Ar. There was a significant effect of bone site for Ct.Th (*p* < 0.0001). Visual inspection of the funnel plots suggested no evidence of publication bias for vBMD, BV/TV, Tb.Th, Tb.N, or Ct.Ar (Figure S10A-D, G). Findings from the Egger regression suggested publication bias was present and a trim-and-fill analysis was performed resulting in five imputed studies for Tb.Sp (Figure S10E), and two imputed studies for Ct.Th (Figure S10F).

#### Other models

The effect of probiotics on bone outcomes was also examined using preclinical models with diabetes,^29^ arthritis,^49^^,63–66^ fracture healing,^67–69^ tenofovir disoproxil fumarate-induced bone loss,^73^ retinoic-induced secondary osteoporosis,^74,75^ CoCrMo particle-induced osteolysis,^76^ ulcerative colitis,^77,78^ dorsal incision surgery,^26^ glucocorticoid-induced osteoporosis,^4^^,70–72^ depression,^79^ cigarette smoking inhalation,^36^ chronic mild stress,^80^ bone cancer pain,^81^ co-intervention with a high-fat diet,^32^^,61,62,88^ or antibiotics,^5^ genetic modification,^7^ as well as an orchidectomy model.^35^ Due to the limited number of studies within each of these conditions, no meta-analyses were conducted.

##### Bone mineral density

In female rats, *L. fermentum* ZS40 and *L. plantarum* HFY15 treatment [6 weeks; 1 × 10^10^ CFU daily] protected against femur bone loss associated with retinoic-induced secondary osteoporosis.^74,75^ In males, *L. plantarum* KRHPS1 [4 weeks; 1 × 10^9^ CFU daily], *L. reuteri* ATCC PTA 6475 [4 weeks; 1 × 10^9^ CFU/mL three times per week^70^] and a medium or high dose of *L. plantarum* LP45 [8 weeks; 1-2 × 10^9^ CFU daily^72^] demonstrated protective bone effects using a glucocorticoid-induced osteoporosis mouse model^71^—VSL#3 had no effect at the femur of male rats [4 weeks; 1 × 10^9^ CFU/mL three times per week^70^]. However, in combination with a high-fat diet, VSL#3 (8 weeks; 1 × 10^9^ CFU/mL every other day) demonstrated greater BMD at the femur in female mice.^61^ No effect was reported in male mice provided *Bifidobacterium pseusocatenulatum* CECT 776 [14 weeks; 1 × 10^9^ CFU/mL daily] or *Lactobacillus coryniformis subsp. Torquens* (10 weeks; 1 × 10^9^ CFU/mL daily) at the femur.^32^^,62^ Furthermore, a bone-promoting probiotic effect at the femur and tibia was observed in a chronic mild stress model using a probiotic mixture of *Bifidobacterium* and *Lactobacillus* species^80^ but not *Faecalibacterium prausnitzii.*^79^ In males, probiotics were protective against bone mineral loss associated with orchidectomy,^35^ diabetes,^29^ arthritis,^49^^,64–66^ and tenofovir disoproxil fumarate^73^ but no effect was observed in male rats exposed to cigarette smoke.^36^ Females were not included in these studies.

##### Trabecular and cortical bone structure

The observed increase in BMD was accompanied by greater BV/TV, Tb.N, and/or Tb.Th as well as lower Tb.Sp.^5^^,^^29^^,^^35^^,^^49^^,61,65,66,68,70–75^ Bone changes were also supported by greater BFR and MAR.^4^^,^^29^^,73^ When BMD did not differ between study groups, there were some positive trabecular bone structure changes,^32^^,^^36^^,62,88^ also with greater BFR and MAR.^88^

### Secondary outcomes

#### Limited studies reporting measures of bone strength

Few studies (*n* = 13) examined the potential protective role of probiotics on bone strength outcomes. The OVX model resulted in significant reductions in bone strength outcomes at the femur and *B. longum* UBBL-64M1395, *L. brevis* AR281, and *L. plantarum* GMNL-662 were able to attenuate these detrimental changes, comparable to SHAM levels,^45^^,60,86^ Compared to the OVX control, *L. rhamnosus* (strain undefined) increased the load, displacement, and energy at the proximal tibia,^57^ while *L. rhamnosus* HN001 had no effect on bone strength at the femur—a large variance among the data was denoted by the authors.^52^ The protective effect on bone strength was less consistent among other rodent models. Interestingly, a dose-dependent effect of *L. plantarum* LP45 on bone strength of the femur was observed in female rats with glucocorticoid-induced osteoporosis.^72^ However, probiotics had no effect on bone strength properties in male mice amid some improvements in bone structure outcomes previously described.^4^^,70^ VSL#3, but not *L. coryniformis subsp. Torquens*, administration increased peak load at the femur in mice consuming a high-fat diet.^61,62^ LGG administration resulted in favorable bone strength changes of tenofovir disoproxil fumarate-induced bone loss at the femur but not tibia in mice post-antibiotics.^5^^,73^ Only one study reported probiotic effects on bone strength outcomes using a model of cigarette smoke exposure, but findings were null.^36^

#### Using serum and bone marrow analytes to probe the potential mechanisms of probiotic-mediated bone modulation

Probiotic supplementation increased the short-chain fatty acid butyrate in the intestines.^7–9^^,^^33^^,80^ In intact rodents, LGG increased butyrate and CD8^+^ regulatory T (Treg) cells in the bone marrow and subsequently upregulated Wnt10b to stimulate bone formation.^7^ This was also shown in rodents fed a high-fat diet and in diabetes.^29^^,^^32^ Probiotic supplementation resulted in higher levels of bone formation markers, including serum procollagen type I N-terminal propeptide or osteocalcin,^3^^,^^7^^,^^24^^,^^32^^,^^34^ but not osteocalcin in the bone marrow.^24^ Compared to the control group, the serum bone resorption marker C-terminal telopeptide of type I collagen (CTx) was lower in mice provided *L. acidophilus*,^34^ but N-terminal telopeptide of type I collagen (NTx) was greater in the probiotic group containing a mixture of *Lactobacillus* and *Bifidobacterium*.^80^

Short-chain fatty acids also reduce proinflammatory T-helper cells (Th17) and decrease osteoclast differentiation. In OVX rats receiving a probiotic supplementation, there was a significant effect on the Th17/Treg ratio in the bone marrow—decreased Th17 and increased CD4^+^CD25^+^Foxp3^+^ Treg cells,^8^^,^^37^^,^^39^^,^^45^^,55^ as well as C19^+^ Breg cells.^45^ Contrary to these findings, there was no probiotic effect on CD4^+^CD25^+^Foxp3^+^ Treg cells using *L. paracasei* DSM 13434 or a *Lactobacillus* mixture,^24^ while *pAKK* reduced Treg cells in the bone marrow in both intact and OVX mice.^23^ Since Th17 cells release pro-inflammatory cytokines, studies subsequently reported decreased serum IL-17, tumor necrosis factor alpha (TNF-α), and/or IL-6,^8^^,^^37–39^^,^^45^^,^^47^ as well as receptor activator of NF-kB ligand (RANKL), TNF-α, and/or IL-17 in the bone marrow.^6^^,^^9^^,^^24^^,^^37–39^^,^^47^^,55,60^ Studies also reported lower serum bone resorption markers (pyridinoline, tartrate-resistant acid phosphatase, CTx, and/or NTx) among OVX rodents provided LGG, *L. gasseri*, or *Lp. plantarum* compared to the control,^3^^,^^25^^,56,60^—other probiotic supplements (*L. paracasei* DSM 13434, *Lactobacillus* mixture, and *L. rhamnosus*) showed no effect on serum tartrate-resistant acid phosphatase and/or CTx.^24^^,57^  *L. plantarum* CJNU 3003, *Lp. Plantarum*, *L. rhamnosus*, *and L. intestinalis* resulted in decreased serum alkaline phosphatase,^27^^,57,58,60,87^ while other bacteria strains had null effects.^23^^,^^27^^,58^ No probiotic effect on serum osteocalcin or osteocalcin in the bone marrow was reported in mice provided *pAKK* or a *Lactobacillus* mixture, respectively.^23^^,^^24^

## Discussion

Probiotic treatment improved outcomes of bone health in some but not all studies using rodent models. The beneficial effects were most prominent in OVX rodent models and align with the findings from clinical studies.^10–13^ With the exception to one study using *Bacillus subtills* C-3102,^10^ these human studies have demonstrated bone supporting effects of probiotics in postmenopausal women using *Lactobacillus* as a single-strain, a mixture of strains or within a multispecies supplement.^11–13^ However, in terms of strain-specific effects, defining specific *Lactobacillus* strains to promote bone health in humans require further investigation. In our systematic review, further analyses on probiotic strains were not performed because many different strains were studied. Findings in intact rodent models were less consistent and depended on the probiotic genus. Other rodent models included a limited number of studies that reported inconsistent effects, so it is difficult to comment on the efficacy of probiotic treatments in these other models. For example, two studies used a model of microbiome dysbiosis via antibiotic treatment [AZM provided to OXV rats^57^ or ampicillin + neomycin provided to male mice^5^] but the antibiotic effect on bone was inconsistent. Specifically, AZM treatment for 4 weeks had a favourable effect on femur histomorphometric structure in OVX rats.^57^ In contrast, ampicillin and neomycin reduced BV/TV at the femur and vertebrae and resulted in lower femur Tb.Th and Tb.N and greater Tb.Sp in male mice.^5^  *L. reuteri* 6475, but not LGG or *L. rhamnosus*, resulted in favourable bone outcomes in these models.^5^^,57^ Of note is that non-pathogenic, non-probiotic *Escherichia coli* was used as a negative control in a few studies that used different models (OVX, administration of tenofovir disproxil fumarate or antibiotics) and was shown to have a null effect on bone outcomes.^3^^,^^5^^,73^

The bone-promoting effects of probiotics are mediated by supporting the optimal state of the gut microbiome, including the mitigation of microbial dysbiosis, intestinal inflammation, and gut permeability.^3–9^ Additionally, probiotic-produced metabolites such as the short-chain fatty acid butyrate are increased in the small intestine and increase the expression of Treg cells in the bone marrow.^7^ This signals an upregulation of Wnt10b expression, which stimulates bone formation through the Wnt signaling pathway in osteoblasts.^7^ In OVX models, Th17-mediated release of IL-6, IL-17A, TNF-α, and RANKL increased osteoclast differentiation.^6^^,^^8^^,^^9^^,^^24^^,^^37–39^^,^^45^^,^^47^^,55,60^ Findings consistently demonstrated that probiotics rebalanced Th17/Treg cells, shown to be skewed during OVX, and thus reduced bone resorption.^8^^,^^37^^,^^39^^,^^45^^,55^ Decreased Th17 cells results in a reduction in pro-inflammatory cytokines in the serum and bone marrow and the downstream effects of probiotics to reduce osteoclast activity or increase osteoblast activity, resulting in higher BMD and more favorable bone structure properties.^8^^,^^37^^,^^39^^,^^45^^,55^ Because probiotics affect bone in a strain-specific manner, formulating the ideal combination of bacteria strains to optimize BMD and strength via the upregulation of bone formation and/or downregulation of bone resorption remains unknown.

There was large heterogeneity among the included studies—particularly for the syntheses of BV/TV (intact, OVX), Tb.Th (intact, OVX), Tb.N (intact, OVX), Tb.Sp (OVX), Ct.Ar (OVX), and Ct.Th (OVX). We explored probiotic genus, bone site of analysis, sex, and rodent type as potential reasons for the observed heterogeneity. Probiotic genus contributed minimally to the heterogeneity, and a lower level of biological classification (ie, species or strain) may be required. Using intact rodents, *Lactobacillus* strains were the most studied probiotics (71% of studies), followed by probiotics containing a combination of *Lactobacillus* and *Bifidobacterium* strains (14% of studies; Table S3). One study investigated a *Bifidobacterium* strain, and few studies investigated other genera either alone or in a probiotic mixture, including *Akkermansia*, *Saccharomyces*, and *Streptococcus*, *Bacillus*, and *Enterococcus* (Table S3). Similar to the intact rodent models, most studies investigated the effects of *Lactobacillus* strains on bone outcomes in OVX rodents (65% of studies), followed by 25% of the studies investigating other genera beyond *Lactobacillus* or *Bifidobacterium* (*Bacillus*, *Akkermansia*, *Saccharomyces*, *Streptococcus*, *Prevotella*, *Rothia*, *Propionibacterium*, *Bacteroides*; Table S4). Three studies used a probiotic containing *Bifidobacterium* strains, and one study investigated a probiotic containing a mixture of *Lactobacillus* and *Bifidobacterium* strains (Table S4). The assessment of bone site, sex, or rodent type subgrouping was limited. The femur, compared to the tibia and spine, was the most studied site. However, the bone outcomes reported were not consistent among all studies and complicate the comparisons. Accordingly, recommendations for a minimum set of bone outcomes to be reported when using μCT have been developed as an attempt to standardize studies and foster transparency.^89^ Of note, DXA was used as an alternative method of quantifying BMD in a few studies,^36^^,52–54,58^ but was not included in the meta-analyses due to differences in the values obtained compared to μCT.^90^ There was limited reporting on functional measures of bone strength outcomes. Two rat studies and two mouse studies reported strength measures, resulting in no effect and greater bone strength with a probiotic intervention, respectively. The difference between the response in rats and mice may be attributed to the different methodology and sizes of the bones. Further investigation on functional measures is warranted as a predictor of fracture. In intact rodents, only one study in rats was included in the meta-analysis, while all other studies were conducted in mice. The opposite is true for OVX studies in which rats rather than mice were predominately studied, with similar responses between species. Sex did not influence the probiotic effect. Most studies included one sex of rodents, while one study allowed for investigating sex-specific effects in which a probiotic treatment resulted in increased BMD and favorable trabecular bone structure changes in healthy male but not female mice.^28^

Interpretation of the current findings should consider the inconsistencies with reporting specific study details, large heterogeneity among studies, and challenges with generalization to humans. Quality assessment using the ARRIVE guidelines^18^ suggested an opportunity to improve the transparency and reporting of intervention studies in animals and consideration of the essential information that has been less commonly reported, including aspects of the study design, sample size calculation, inclusion and exclusion criteria, randomization methods, blinding, defining outcome measures, and assumptions considered in the statistical analysis. The lack of reporting from the individual studies influenced the risk of bias assessment, as demonstrated by the “unclear” ratings for all items.

While this review focused on probiotics in the form of isolated bacteria strain(s), future study can include fermented food products containing probiotics. When foods are fermented with bacteria, it can become a source of probiotics—though not all fermented foods contain probiotics. While fermentation can increase the shelf-life of foods, it has quickly regained interest in the dietary landscape with emerging evidence suggesting a positive influence on the gut microbiome and human health.^91^ Because the fermentation process changes the nutritional composition of food products and presents potential nutrient interactions, this area requires future study.

The overall findings from rodent models suggest bone-promoting effects of probiotics, particularly for attenuating OVX-induced bone loss, while specifics regarding the optimal probiotic strain(s), dose, source, and frequency of exposure can be elucidated in future investigations. These findings can inform the design of future clinical trials studying probiotic supplementation.

## Supplementary Material

FINAL_Supplemental_Material_(2)_Yumol_zjae187

## Data Availability

The data that support the findings of this study are openly available in the Brock University Dataverse of Borealis at https://doi.org/10.5683/SP3/LJEMOC.
