# Supplementary material for: A systematic review and meta-analysis of the effects of probiotics on bone outcomes in rodent models
Source: J Bone Miner Res. 2024 Nov 15;40(1):100–13. doi: 10.1093/jbmr/zjae187 (PMC11700591; doi:10.1093/jbmr/zjae187)
Supplement: FINAL_Supplemental_Material_(2)_Yumol_zjae187 [file final_supplemental_material_(2)_yumol_zjae187.docx]

**SUPPLEMENTAL MATERIAL**

**References (continued)**

51. Parvaneh M, Karimi G, Jamaluddin R, Ng AMH, Ibrahim Z, Muhammad SI. *Lactobacillus helveticus* (ATCC 27558) upregulates Runx2 and Bmp2 and modulates bone mineral density in ovariectomy-induced bone loss rats. Clin Interv Aging. 2018 Aug;Volume 13:1555–64.

52. Kruger MC, Fear A, Chua WH, Plimmer GG, Schollum LM. The effect of *Lactobacillus rhamnosus* HN001 on mineral absorption and bone health in growing male and ovariectomized female rats. Dairy Sci Technol. 2009 May;89(3–4):219–31.

53. Gholami A, Dabbaghmanesh MH, Ghasemi Y, Talezadeh P, Koohpeyma F, Montazeri-Najafabady N. Probiotics ameliorate pioglitazone-associated bone loss in diabetic rats. Diabetol Metab Syndr. 2020 Dec;12(1):78.

54. Montazeri-Najafabady N, Ghasemi Y, Dabbaghmanesh MH, Talezadeh P, Koohpeyma F, Gholami A. Supportive role of probiotic strains in protecting rats from ovariectomy-induced cortical bone loss. Probiotics Antimicrob Proteins. 2019 Dec;11(4):1145–54.

55. Guo M, Liu H, Yu Y, Zhu X, Xie H, Wei C, et al. Lactobacillus rhamnosus GG ameliorates osteoporosis in ovariectomized rats by regulating the Th17/Treg balance and gut microbiota structure. GUT MICROBES. 2023 Dec 31;15(1).

56. Lee SH, Lim TJ, Yun EJ, Kim KH, Lim S. Anti-Menopausal Effect of Soybean Germ Extract and Lactobacillus gasseri in the Ovariectomized Rat Model. Nutrients. 2023 Oct 15;15(20):4485.

57. Cegiela U, Londzin P, Janas A, Pytlik M, Folwarczna J. Effect of Administration of Azithromycin and/or Probiotic Bacteria on Bones of Estrogen-Deficient Rats. PHARMACEUTICALS. 2022 Aug;15(8).

58. Gholami A., Dabbaghmanesh M.H., Ghasemi Y., Koohpeyma F., Talezadeh P., Montazeri-Najafabady N. The ameliorative role of specific probiotic combinations on bone loss in the ovariectomized rat model. BMC Complement Med Ther. 2022;22(1):241.

59. Jin E.-S., Kim J.Y., Yang J.-M., Kim J.-S., Min J., Jeon S.R., et al. The Effect of Genetically Modified Lactobacillus plantarum Carrying Bone Morphogenetic Protein 2 Gene on an Ovariectomized Rat. J Korean Neurosurg Soc. 2022;65(2):204–14.

60. Tsai WH, Lin WC, Chou CH, Yang LC. The probiotic *Lactiplantibacillus plantarum* attenuates ovariectomy-induced osteoporosis through osteoimmunological signaling. Food Funct. 2023;14(15):6929–40.

61. Behera J, Ison J, Voor MJ, Tyagi N. Probiotics stimulate bone formation in obese mice via histone methylations. Theranostics. 2021;11(17):8605–23.

62. Song W, Bai Y, Hu J, Li L, He W, Liu C, et al. Lactobacillus coryniformis subsp. torquens inhibits bone loss in obese mice via modification of the gut microbiota. FOOD Funct. 2023 May 22;14(10):4522–38.

63. Sophocleous A., Azfer A., Huesa C., Stylianou E., Ralston S.H. Probiotics Inhibit Cartilage Damage and Progression of Osteoarthritis in Mice. Calcif Tissue Int. 2023;112(1):66–73.

64. Rovenský J, Švík K, Maťha V, Ištok R, Kamarád V, Ebringer L, et al. Combination treatment of rat adjuvant-induced arthritis with methotrexate, probiotic bacteria *Enterococcus faecium*, and Selenium. Ann N Y Acad Sci. 2005 Jun;1051(1):570–81.

65. Lin YY, Chang SLY, Liu SC, Achudhan D, Tsai YS, Lin SW, et al. Therapeutic effects of live *Lactobacillus plantarum* GKD7 in a rat model of knee osteoarthritis. Nutrients. 2022 Aug 1;14(15):3170.

66. Chang SLY, Lin YY, Liu SC, Tsai YS, Lin SW, Chen YL, et al. Oral administration of *Clostridium butyricum* GKB7 ameliorates signs of osteoarthritis in rats. Cells. 2022 Jul 11;11(14):2169.

67. Roberts J, Golloshi M, Harding D, Conduah M, Liu G, Drissi H. Bifidobacterium longum supplementation improves age-related delays in fracture repair. AGING CELL. 2023 Apr;22(4).

68. Roberts JL, Liu G, Darby TM, Fernandes LM, Diaz-Hernandez ME, Jones RM, et al. *Bifidobacterium adolescentis* supplementation attenuates fracture-induced systemic sequelae. Biomed Pharmacother. 2020 Dec;132:110831.

69. Wang Y, Agenor A, Clement A, Hopfgartner A, Whyne C, Nam D. Probiotics: Can it modulate fracture healing? PLOS ONE. 2023 Aug 31;18(8).

70. Chargo NJ, Schepper JD, Rios‐Arce N, Kang HJ, Gardinier JD, Parameswaran N, et al. *Lactobacillus Reuteri* 6475 prevents bone loss in a clinically relevant oral model of glucocorticoid‐induced osteoporosis in male CD‐1 mice. JBMR Plus. 2023 Dec;7(12):e10805.

71. Li S, Han X, Liu N, Chang J, Liu G, Hu S. *Lactobacillus plantarum* attenuates glucocorticoid-induced osteoporosis by altering the composition of rat gut microbiota and serum metabolic profile. Front Immunol. 2024 Jan 9;14:1285442.

72. Jiang X, Qi X, Xie C. *Lactobacillus plantarum* LP45 inhibits the RANKL/OPG signaling pathway and prevents glucocorticoid-induced osteoporosis. Food Nutr Res [Internet]. 2023 Mar 24 [cited 2024 Apr 23];67. Available from: https://foodandnutritionresearch.net/index.php/fnr/article/view/9064

73. Liu H, Gu R, Li W, Zhou W, Cong Z, Xue J, et al. *Lactobacillus rhamnosus* GG attenuates tenofovir disoproxil fumarate-induced bone loss in male mice *via* gut-microbiota-dependent anti-inflammation. Ther Adv Chronic Dis. 2019 Jan;10:204062231986065.

74. Liu X, Zheng J, Li F, Yi R, Mu J, Tan F, et al. *Lactobacillus plantarum* HFY15 helps prevent retinoic acid-induced secondary osteoporosis in wistar rats. Evid Based Complement Alternat Med. 2020 Sep 23;2020:1–10.

75. Liu X, Fan J, Hu J, Li F, Yi R, Tan F, et al. *Lactobacillus fermentum* ZS40 prevents secondary osteoporosis in Wistar Rat. Food Sci Nutr. 2020 Sep;8(9):5182–91.

76. Wang Z, Xue K, Bai M, Deng Z, Gan J, Zhou G, et al. Probiotics protect mice from CoCrMo particles-induced osteolysis. Int J Nanomedicine. 2017 Jul;Volume 12:5387–97.

77. Xu Y, Xie Q, Zhang W, Zhu M, Chen X, Guo D, et al. *Lactobacillus plantarum* GMNL-662 and *Lactobacillus plantarum* 299v prevent osteoporosis in mice with colitis by down-regulating *Akkermansia* in the gut microbiome. J Funct Foods. 2022 Dec;99:105328.

78. Lan H, Liu WH, Zheng H, Feng H, Zhao W, Hung WL, et al. *Bifidobacterium lactis* BL-99 protects mice with osteoporosis caused by colitis *via* gut inflammation and gut microbiota regulation. Food Funct. 2022;13(3):1482–94.

79. Hao Z, Wang W, Guo R, Liu H. *Faecalibacterium prausnitzii* (ATCC 27766) has preventive and therapeutic effects on chronic unpredictable mild stress-induced depression-like and anxiety-like behavior in rats. Psychoneuroendocrinology. 2019 Jun;104:132–42.

80. Lee Y, Oh H, Jo M, Cho H, Park Y. Synergistic effect of n-3 PUFA and probiotic supplementation on bone loss induced by chronic mild stress through the brain–gut–bone axis. J Funct Foods. 2023 Jan;100:105363.

81. Yuan W, Xiao J, Liao H, Xie Z, Zhao Y, Li C, et al. *Lactobacillus rhamnosus* GG and butyrate supplementation in rats with bone cancer reduces mechanical allodynia and increases expression of μ-opioid receptor in the spinal cord. Front Mol Neurosci. 2023 Jun 14;16:1207911.

82. Lee CC, Liao YC, Lee MC, Lin KJ, Hsu HY, Chiou SY, et al. *Lactobacillus plantarum* TWK10 attenuates aging-associated muscle weakness, bone loss, and cognitive impairment by modulating the gut microbiome in mice. Front Nutr. 2021 Oct 13;8:708096.

83. Thompson DD, Simmons HA, Pirie CM, Ke HZ. FDA guidelines and animal models for osteoporosis. Bone. 1995 Oct;17(4):S125–33.

84. Jhong JH, Tsai WH, Yang LC, Chou CH, Lee TY, Yeh YT, et al. Heat-killed *Lacticaseibacillus paracasei* GMNL-653 exerts antiosteoporotic effects by restoring the gut microbiota dysbiosis in ovariectomized mice. Front Nutr. 2022 Feb 4;9:804210.

85. Yuan Y, Yang J, Zhuge A, Li L, Ni S. Gut microbiota modulates osteoclast glutathione synthesis and mitochondrial biogenesis in mice subjected to ovariectomy. Cell Prolif. 2022 Mar;55(3):e13194.

86. Yu J, Hang Y, Sun W, Wang G, Xiong Z, Ai L, et al. Anti-osteoporotic effect of *Lactobacillus brevis* AR281 in an ovariectomized mouse model mediated by inhibition of osteoclast differentiation. Biology. 2022 Feb 24;11(3):359.

87. Jin E, Kim J, Yang J, Kim J, Min J, Jeon SR, et al. The effect of genetically modified *Lactobacillus plantarum* carrying bone morphogenetic protein 2 gene on an ovariectomized rat. J Korean Neurosurg Soc. 2022 Mar 1;65(2):204–14.

88. Eaimworawuthikul S, Tunapong W, Chunchai T, Suntornsaratoon P, Charoenphandhu N, Thiennimitr P, et al. *Lactobacillus paracasei* HII01, xylooligosaccharide and synbiotics improve tibial microarchitecture in obese-insulin resistant rats. J Funct Foods. 2019 Aug;59:371–9.

89. Bouxsein ML, Boyd SK, Christiansen BA, Guldberg RE, Jepsen KJ, Müller R. Guidelines for assessment of bone microstructure in rodents using micro-computed tomography. J Bone Miner Res. 2010 Jun 7;25(7):1468–86.

90. Briggs AM, Perilli E, Parkinson IH, Wrigley TV, Fazzalari NL, Kantor S, et al. Novel assessment of subregional bone mineral density using DXA and pQCT and subregional microarchitecture using micro-CT in whole human vertebrae: Applications, methods, and correspondence between technologies. J Clin Densitom. 2010 Apr;13(2):161–74.

91. Melini F, Melini V, Luziatelli F, Ficca AG, Ruzzi M. Health-promoting components in fermented foods: An up-to-date systematic review. Nutrients. 2019 May 27;11(5):1189.

Table S1. Full search strategy for MEDLINE (Ovid), Embase (Ovid), and CINAHL Complete (EBSCOhost), Dissertations & Theses (PROQUEST), and Web of Science (Core Collection).

|  | MEDLINE  (OVID) | Embase  (OVID) | CINAHL Complete  (EBSCO) | Dissertations & Theses (PROQUEST) | Web of Science  (Core Collection) |
| --- | --- | --- | --- | --- | --- |
| **Concept 1:**  Probiotic | **Headings:**  Probiotics/ or Synbiotics/  Lactobacillus/ or Bifidobacterium/  exp "fermented foods and beverages"/  **Keywords and phrases:**  (probiotic* or lactobac* or bifido* or yeast or synbiotic* or ferment*).mp. | **Headings:**  Probiotic agent/ or  Synbiotic agent/  Lactobacillus/ or Bifidobacterium/  exp fermented product/  **Keywords and phrases:**  (probiotic* or lactobac* or bifido* or yeast or synbiotic* or ferment*).mp. | **Headings:**  (MH “Probiotics”) OR (MH “Lactobacillus”) OR (MH “Bifidobacterium”) OR (MH “Foods, Fermented”)  **Keywords and phrases:**  TX ((probiotic* or lactobac* or bifido* or yeast or synbiotic* or ferment*)) | **Headings: X**  **Keywords and phrases:**  TIABSU(probiotic* or lactobac* or bifido* or yeast or synbiotic* or ferment*) | **Headings: X**  **Keywords and phrases:**  ALL=(probiotic* or lactobac* or yeast or synbiotic* or ferment*) |
| **Concept 2:**  Bone | **Headings:**  exp "Bone and Bones"/ Osteology/ exp Osteoporosis/ Bone Density/  **Keywords and phrases:**  ((bone* and (tissue* or density or mineral or structure* or microarchitect* or histomorphometr* or loss or formation or surface or cancellous or spongy or trabecular or cortical or volume or compact)) or (cortical and (thickness or area or width)) or (trabecular and (number or thickness or separation or width)) or mineral apposition rate or osteolog*).mp. | **Headings:**  exp Bone/  Osteology/ exp Osteoporosis/  Bone Density/ exp bone microarchitecture/ or exp bone structure/  **Keywords and phrases:**  ((bone* and (tissue* or density or mineral or structure* or microarchitect* or histomorphometr* or loss or formation or surface or cancellous or spongy or trabecular or cortical or volume or compact)) or (cortical and (thickness or area or width)) or (trabecular and (number or thickness or separation or width)) or mineral apposition rate or osteolog*).mp. | **Headings:**  (MH "Bone and Bones+") OR (MH “Osteoporosis”) OR  (MH "Bone Density")  **Keywords and phrases:**  TI (((bone* and (tissue* or density or mineral or structure* or microarchitect* or histomorphometr* or loss or formation or surface or cancellous or spongy or trabecular or cortical or volume or compact)) or (cortical and (thickness or area or width)) or (trabecular and (number or thickness or separation or width)) or “mineral apposition rate” or osteolog*)) OR AB ( ((bone* and (tissue* or density or mineral or structure* or microarchitect* or histomorphometr* or loss or formation or surface or cancellous or spongy or trabecular or cortical or volume or compact)) or (cortical and (thickness or area or width)) or (trabecular and (number or thickness or separation or width)) or “mineral apposition rate” or osteolog*)) | **Headings: X**  **Keywords and phrases:**  TIABSU((bone* and (tissue* or density or mineral or structure* or microarchitect* or histomorphometr* or loss or formation or surface or cancellous or spongy or trabecular or cortical or volume or compact)) or (cortical and (thickness or area or width)) or (trabecular and (number or thickness or separation or width)) or “mineral apposition rate” or osteolog*) | **Headings: X**  **Keywords and phrases:**  (TI=(((bone* and (tissue* or density or mineral or structure* or microarchitect* or histomorphometr* or loss or formation or surface or cancellous or spongy or trabecular or cortical or volume or compact)) or (cortical and (thickness or area or width)) or (trabecular and (number or thickness or separation or width)) or mineral apposition rate or osteolog*))) OR AB=(((bone* and (tissue* or density or mineral or structure* or microarchitect* or histomorphometr* or loss or formation or surface or cancellous or spongy or trabecular or cortical or volume or compact)) or (cortical and (thickness or area or width)) or (trabecular and (number or thickness or separation or width)) or mineral apposition rate or osteolog*)) |
| **Concept 3:**  Rodent | **Headings:**  *rodentia/ or exp guinea pigs/ or *muridae/ or exp cricetinae/ or exp murinae/ or exp mice/ or exp rats/  **Keywords and phrases:**  (rodent* or mouse or mice or mus or rat or rats or guinea pig* or cricetinae or hamster*).mp. | **Headings:**  exp Rodent/ or exp Rodent model/ or exp guinea pig/ or *muridae/ or hamster/ or exp murinae/ or exp mouse/ or exp rat/  **Keywords and phrases:**  (muridae or murinae or rodent* or mouse or mice or mus or rat or rats or guinea pig* or cricetinae or hamster*).mp. | **Headings:**  (MH “Rodents+”) OR (MH “Guinea pigs”) OR (MH "Hamsters") OR (MH "Mice") OR (MH "Rats")  **Keywords and phrases:**  TI ((muridae or murinae or rodent* or mouse or mice or mus or rat or rats or “guinea pig*” or cricetinae or hamster*)) OR AB ((muridae or murinae or rodent* or mouse or mice or mus or rat or rats or “guinea pig*” or cricetinae or hamster*)) | **Headings: X**  **Keywords and phrases:**  TIABSU(muridae or murinae or rodent* or mouse or mice or mus or rat or rats or “guinea pig*” or cricetinae or hamster*) | **Headings: X**  **Keywords and phrases:**  (TI=((muridae or murinae or rodent* or mouse or mice or mus or rat or rats or “guinea pig*” or cricetinae or hamster*) )) OR AB=((muridae or murinae or rodent* or mouse or mice or mus or rat or rats or “guinea pig*” or cricetinae or hamster*)) |

Table S2. Summary of risk of bias assessment guidelines (Bott et al., 2022).

|  | Yes | No | Unclear |
| --- | --- | --- | --- |
| Selection bias | | | |
| Sequence generation | Description of the random component in the allocation sequence generation. | No mention of sequence allocation. | When randomization was mentioned but not specified how the allocation sequence was generated. |
| Baseline characteristics | When individual animals were equally distributed by body weight between study groups at baseline. | No body weight data was reported. | When baseline body weight was reported but distribution between study groups was not mentioned. |
| Allocation concealment | When sufficient detail was provided on the method for concealing animal intervention allocation. | * | When no information was provided. |
| Performance bias | | | |
| Random housing | When animals were randomly housed during the study. | * | When no information was provided. |
| Blinding | When sufficient detail regarding the measures taken to blind the caregivers and researchers from the animal intervention groups. | * | When no information was provided. |
| Detection bias | | | |
| Random outcome assessment | When animals were randomly selected for outcome assessment. | * | When no information was provided. |
| Blinding | When researchers were blinded during analysis from animal intervention groups. | * | When no information was provided. |
| Attrition bias | | | |
| Incomplete outcome data | When the sample size between the methods and results was clearly explained. | When the sample size did not match between the methods and results with no explanation. | When the sample size matched between the methods and results with no explanation. |
| Reporting bias | | | |
| Selective outcome reporting | When details regarding outcome reporting were selected and scored. | * | When no explanation was included. |
| Other | | | |
| Other sources of bias | When any other potential sources of bias were discussed in sufficient detail. | * | When no other information was discussed. |

**If a response of ‘yes’ could not be identified from the information provided in the paper, it was assumed that these aspects were considered in some way. Therefore, an assessment of ‘unclear’ was identified for these criteria.*

Table S3. Intact rodent model: Summary of findings for the primary bone outcomes.

| **Country [ref]** | **Rodent species (sex; age)** | **Probiotic intervention** | **Probiotic dose (frequency)** | **Study duration** | **Intervention groups (n)** | **Bone outcomes (bone site of analysis)** |
| --- | --- | --- | --- | --- | --- | --- |
| Untied States [McCabe 2013] | C57BL/6 mice  (M; 14 weeks) | *L. reuteri* ATCC PTA 6475 (LBR) | 0.3 mL of 1 x 10^9^ CFU/mL  *(three times per week)* | 1 week acclimatization  4 weeks intervention | CON (10) LBR (10) | ↑ vBMD, BMC, BV/TV, Tb.N, Tb.Th (femur, vertebrae) ↓ Tb.Sp (femur, vertebrae) - Tt.Ar, Ct.Ar, Ma.Ar, Ct.Ar/Tt.Ar, Ct.Th (femur) ↑ BFR (femur) |
|  | C57BL/6 mice  (F; 14 weeks) | *L. reuteri* ATCC PTA 6475 (LBR) | 0.3 mL of 1 x 10^9^ CFU/mL (*three times per week)* | 1 week acclimatization  4 weeks intervention | CON (10) LBR (10) | - BV/TV (femur, vertebrae) |
| Untied States [Zhang 2015] | C57BL/6 mice  (M; 14 weeks) | *L. reuteri* ATCC PTA 6475 (LBR) | 0.3 mL of 1 x 10^9^ CFU/mL (*three times per week)* | 1 week acclimatization  4 weeks intervention | CON (10) LBR (10) | - vBMD, BMC, BV/TV, Tb.N, Tb.Th, Tp.Sp (femur) - Tt.Ar, Ct.Ar, Ma.Ar, Ct.Ar/Tt.Ar, Ct.Th, Ec.Pm, Ps.Pm (femur) ↑ ObS (femur) - OcS, MAR (femur) |
| Untied States [Collins 2019] | C57BL/6 mice  (M; 12 weeks) | *L. reuteri* ATCC PTA 6475 (LBR) | 3.3 x 10^8^ CFU/mL *(daily)* | 1 week acclimatization  4 weeks intervention | CON (10) LBR (10) | ↑ vBMD, BV/TV, Tb.Th (femur) ↓ Ma.Ar, P.Pm (femur) - TMD, BMC, Tb.N, Tb.Sp, Ct.Ar, Ct.Th, Ec.Pm, MOI (femur) - BFR, MAR (femur) |
| Untied States [Blanton 2018] | ND4 Swiss Webster retired breeder mice  (M; 34-35 weeks) | *B. pseudocatenulatum* CECT 7765 (BBP) | 1 x 10^8^  CFU/g diet *(daily)* | 8-9 weeks acclimatization  26 weeks intervention | CON (3) BBP (5) | - BV/TV, SA/BV, Tb.Th, Tb.N, Tb.Sp, Tb. Patt. Fact (tibia, femur) - Ct.Th, Ma.Ar (tibia, femur) |
| Spain [Fernandez-Murga 2020] | C57BL/6 mice  (M; 6-8 weeks) | *B. pseudocatenulatum* CECT 7765 (BBP) | 1 x 10^9^ CFU/mL *(daily)* | 1 week acclimatization  14 weeks   intervention | CON (10) BBP (10) | - vBMD, BV/TV, Tb.N, Tb.Th, Tb.Pf, Tb.Sp (femur) - TMD (femur) |
| Taiwan [Lee 2021] | Institute of Cancer Research mice (M; young (Y), 17 weeks; aged (A), 82-95 weeks) | *Lactiplantibacillus plantarum* (TWK10) | 1 x 10^9^ CFU/mL *(daily)* | acclimatization period ND  8 weeks intervention | Y-CON (8) Y-TWK10 (9)  A-CON (9)  A-TWK10 (7) | Young:  - vBMD, Tb.Th (femur) ↑ BV/TV, Tb.N (femur) ↓ Tb.Sp (femur)  Aged: - vBMD, Tb.Th, Tb.Sp (femur) ↑ BV/TV, Tb.N (femur) |
| China [Zhai 2023] | SAMP6 mice (M; 7 weeks) | *L. acidophilus* ATCC 4356 (LAS) | 400 µL, CFU ND *(daily)* | acclimatization period ND  8 weeks intervention | CON (6) Heat-activated LAS (6)  Inactive LAS (6) | Heat-activated LAS: ↑ BV/TV, Tb.N (femur) ↓ Tb.Sp (femur) - Tb.Th (femur)  Inactive LAS: - BV/TV, Tb.Th, Tb.N, Tb.Sp (femur) |
| Sweden [Lawenius 2022] | C57BL/6J mice (M; 10 weeks) | *Lacticaseibacillus paracasei* DSM13434*, Lactiplantibacillus plantarum* DSM 15312 and DSM 15313 (LMIX) | 1 x 10^9^ CFU/mL *(daily)* | acclimatization period ND  6 weeks intervention (starting 2 weeks before surgery) | CON (15) LMIX (15) | - vBMD, Ct.Ar (femur); BV/TV, Tb.N, Tb.Sp, Tb.Th (L5); aBMD (whole body) |
| United States [Collins 2016] | Balb/c (F; 11 weeks) | *L. reuteri* ATCC PTA 6475 (LBR) | 1 x 10^9^ CFU/mL *(three times per week)* | 1 week acclimatization  8 weeks intervention | CON (7) LBR (7) | - TMD, BMC, BV/TV, Tb.N, Tb.Th, Tb.Sp, BFR, MAR, Ec.Pm, Ps.Pm, Ma.Ar, Ct.Ar, Tt.Ar (femur) |
| Untied States [Tyagi 2018] | C57BL/6 mice  (F; 10 weeks) | *L. rhamnosus* GG (LGG) | 1 x 10^9^ CFU/mL *(daily)* | acclimatization period ND  4 weeks intervention | Irr. Ab (10) Irr. Ab+LGG (10) | Week 2: ↑ BV/TV (spine)  Week 4: ↑ BV/TV (spine, femur) ↑ MAR, BFR/BS (femur) - OcN/BS, OcS/BS (femur) |
| Untied States [Li 2016] | C57BL/6J mice  (F; 10 weeks) | *L. rhamnosus* GG (LGG)  VSL#3 (*B. breve, B. longum, B. infantis, L. acidophilus, L. plantarum, L. paracasei, L. bulgaricus,* *S. thermophilus*) | 1 x 10^9^ CFU/mL *(twice per week)* | acclimatization period ND  4 weeks intervention | SHAM (10) SHAM+LGG (10) SHAM+VSL#3 (10) | LGG: ↑ BV/TV (femur) - BV/TV (spine) - Ct.Ar (femur)  VSL#3: - BV/TV (spine, femur) - Ct.Ar (femur) |
| Sweden [Ohlsson 2014] | C57BL/6N mice (F; 6 weeks) | *L. paracasei* DSM 13434 (LBP) *L.* Mixture, LMIX (*L. paracasei* DSM 13434, *L. plantarum* DSM 15312 and DSM 15313) | 1 x 10^8^ CFU/mL *(daily)* | 2 weeks acclimatization  6 weeks intervention | SHAM (6) SHAM+LBP (6) SHAM+LMIX (6) | - vBMD, BV/TV, Tb.Th, Tb.N, Tb.Sp (femur) - BMC, Ct.Ar, Ct.Th, Tt.Ar (femur) |
| Sweden [Lawenius 2020] | C57BL/6 mice  (F; 12 weeks) | pasturized *A. muciniphila* (p*AKK*) | 2 x 10^8^ CFU *(daily)* | acclimatization period ND  4 weeks intervention | SHAM (8) SHAM+p*AKK* (8) | ↓ vBMD (femur); Tb.Th (vertebra) ↓ Ct.Ar (vertebra) - BV/TV, Tb.Th, Tb.N, Tb.Sp (femur); BV/TV, Tb.N, Tb.Sp (vertebra) - Ct.Th, Ct.Ar (femur); Ct.Th (vertebra) |
| United States [Madel 2023] | C57BL/6 mice (F; 6 weeks) | *Saccharomyces boulardii* (Sb) | 3 g/kg bodyweight CFU ND *(three times per week)* | 6 weeks intervention | SHAM (14) SHAM+Sb (11) | - BV/TV, Tb.N, Tb.Sp, Tb.Th (femur) |
| China [Jia 2021] | Sprague-Dawley rats  (F; 10 weeks) | Lifespace, lsPro (*L. rhamnosus* HN001, *B. lactis* BI-04, *B. animals* HN019, *L. fermentum* SBS-1, *L. reuteri* 1e1, *B. longum* BB536, *B. breve* M16-V, *B. infantis* Bi-26, *L. paracasei* Lpc-37) | 1 x 10^7^ CFU *(daily)* | 1 week acclimatization  7 weeks intervention | SHAM (5) SHAM+lsPro (5) | - BV/TV, Tb.N, Tb.Pf, Tb.Sp (femur) |
| Brazil [Tribst 2019] | Wistar rats (M; 3 weeks) | Mixture, MIX (*L. acidophilus, E. faecium, B. subtilis,* and*B. bifidum*) | 2 x 10^9^ CFU/kg *(daily)* | 1 week acclimatization  26 weeks intervention | CON (8) MIX (8) | ↑ area, Tb.Th proximal (femur) - aBMD, BMC, Tb.Th medial and distal (femur) |

ABL, alveolar bone loss; aBMD, areal bone mineral density; BFR, bone formation rate; BMC, bone mineral content; BV/TV, bone volume fraction; CON, control; Ct.Ar, cortical area; Ct.Ar/Tt.Ar, cortical area fraction; Ct.Th, cortical thickness; Ma.Ar, medullary area; MAR, mineral apposition rate; ND, not defined; OcN/BS, number of osteoclasts per bone surface; ObS, osteoblast surface; OcS, osteoclast surface; SHAM, sham-operated (control); Tb.N, trabecular number; Tb.Pf, trabecular pattern factor; Tb.Sp, trabecular separation; Tb.Th, trabecular thickness; TMD, tissue mineral density; Tt.Ar, total area; vBMD, volumetric bone mineral density. ↑ denotes the bone outcome is greater in the probiotic group compared to the control group; ↓ denotes the bone outcomes is lower in the probiotic group compared to the control group; - denotes there is no difference between the probiotic group and the control group.

Table S4. Ovariectomized (OVX) rodent model: Summary of findings for the primary bone outcomes.

| **Country [ref]** | **Rodent species (sex; age)** | **Probiotic intervention** | **Probiotic dose (frequency)** | **Study duration** | **Intervention groups (n)** | **Bone outcomes (bone site of analysis)** |
| --- | --- | --- | --- | --- | --- | --- |
| India  [Dar 2018, nutr] | BALB/c mice (F; 8-10 weeks) | *B. clausii* (BBC) | 0.2 mL of 1 x 10^9^ CFU/mL *(daily)* | acclimatization period ND  6 weeks intervention | OVX (10) OVX+BBC (10) | ↑ vBMD, BV/TV, Tb.Th, Tb. N, Conn.Dn (femur, tibia, L5) ↓ Tb.Sp, Tb.Pf (femur, tibia, L5) ↑ TMD, Tt.Ar, T.Pm, Ct.Ar, Ps.Pm, Ct.Th (femur, tibia) |
| India  [Dar 2018] | BALB/c mice (F; 8-10 weeks) | *L. acidophilus* ATCC 4356 (LBA) | 0.2 mL of 1 x 10^9^ CFU/mL *(daily)* | acclimatization period ND  6 weeks intervention | OVX (10) OVX+LBA (10) | ↑ vBMD, BV/TV, Tb.Th, Tb. N, Conn.Dn (femur, tibia, L5) ↓ Tb.Sp, Tb.Pf (femur, tibia, L5) ↑ TMD, Tt.Ar, T.Pm, Ct.Ar, Ps.Pm, Ct.Th (femur, tibia) |
| India  [Sapra 2021] | BALB/c mice (F; 8-10 weeks) | *L. rhamnosus* UBLR-58 MTCC 5402 (LR) | 0.4 mL of 1 x 10^9^ CFU/mL *(daily)* | acclimatization period ND  6 weeks intervention | OVX (6) OVX+LR (6) | ↑ vBMD, BV/TV, Tb.Th (femur, tibia, L5) ↓ Tb.Sp (femur, tibia, L5) ↑ Tt.Ar, T.Pm (femur); TMD, Tt.Ar (tibia) ↓ Ct.Po (femur, tibia) - T.Pm (tibia); TMD (femur) |
| China  [Yang 2020] | Institute of Cancer Research mice (F; 8 weeks) | *L. plantarum* GKM3 (GKM3) *Lactobacillus paracasei* GKS6 (GKS6) | 2 x 10^11^ CFU/g *(daily)* | 1 week acclimatization  4 weeks treatment | OVX (9) OVX+GKM3 (9) OVX+GKS6 (9) | GKM3: ↓ Tb.Sp (femur) - vBMD, BV/TV, Tb.Th, Tb.N (femur)  GKS6: ↑ vBMD, Tb.Th (femur) ↓ Tb.Sp (femur) - BV/TV, Tb.N (femur) |
| Taiwan [Jhong 2022] | Institute of Cancer Research mice (F; 8 weeks) | *L. paracasei* GMNL-653 (dead)  *L. paracasei* GMNL-678 | 1 x 10^10^ CFU/mL *(daily)* | acclimatization period ND  4 weeks intervention | OVX (8)  OVX+GML-678 (8)  OVX+GMNL-653 (8) | GML-678: ↑ vBMD, BV/TV (tibia)  GMNL-653: ↑ vBMD, BV/TV (tibia) |
| Sweden [Lawenius 2020] | C57BL/6 mice (F; 12 weeks) | pasturized *A. muciniphila* (p*AKK*) | 2 x 10^8^ CFU *(daily)* | acclimatization period ND  4 weeks intervention | OVX (8) OVX+ p*AKK* (8) | ↓ vBMD, BV/TV (femur) ↓ Cr.Ar (femur) - Tb.N, Tb.Sp, Tb.Th (femur); BV/TV, Tb.N, Tp.Sp, Tb.Th (vertebra) -Ct.Th (femur, vertebra); Ct.Ar (vertebra) |
| Sweden [Ohlsson 2014] | C57BL/6N mice (F; 6 weeks) | *L. paracasei* DSM 13434 (LBP) *L.* mixture, LMIX (*L. paracasei* DSM 13434, *L. plantarum* DSM 15312 and DSM 15313) | 1 x 10^8^ CFU/mL *(daily)* | 2 weeks acclimatization  6 weeks intervention | OVX (6) OVX+LBP (6) OVX+LMIX (6) | LBP*:* ↑ BMC, Ct.Ar (femur) - vBMD, BV/TV, Tb.Th, Tb.N, Tb.Sp (femur) - Tt.Ar, Ct.Th (femur)  LMIX*:* ↑ BMC, Ct.Th (femur) - vBMD, BV/TV, Tb.Th, Tb.N, Tb.Sp (femur) - Tt.Ar (femur) |
| Sweden [Ohlsson 2021] | C57BL/6N mice (F; 9 weeks) | *L.* mixture, LMIX (*L. paracasei* DSM 13434, *L. plantarum* DSM 15312 and DSM 15313) | 1 x 10^8^ CFU/mL *(daily)* | 3 weeks acclimatization  1.5 week surgery  5.5 week intervention | OVX (7) OVX+LMIX (7) | ↑ Tb.Th (tibia) - BV/TV, Tb.N (tibia) - Ct.Ar, Ct.Th (tibia) |
| United States [Britton 2014] | BALB/c mice (F; 12 weeks) | *L. reuteri* ATCC PTA 6475 (LBR) | 0.3 mL of 1 x 10^9^ CFU/mL *(three times per week)* | 1 week acclimatization  4 weeks intervention | OVX (8) OVX+LBR (8) | ↑ vBMD, BMC, BV/TV, Tb.N (femur) ↓ Tb.SP (femur) - vBMD, BMC, BV/TV, Tb.N, Tb.Th, Tb.Sp (vertebrae); Tb.Th (femur) - Tt.Ar, Ct.Ar, Ma.Ar, Ct.Ar/Tt.Ar,Ct.Th, Ps.Pm, Ec.Pm (femur) - BFR, ObS (vertebrae) |
| China  [Yuan 2021] | C57BL/6 mice (F; 6 weeks) | *B. vulgatus* ATCC 8482 (BV) | 0.1 mL of 5 x 10^9^ CFU/mL *(every two days)* | 1 week acclimatization  1 week postoperative  recovery  8-9 weeks intervention | OVX (5) OVX+BV (7) | ↑ vBMD (L2-L5); BV/TV, BS/TV, Tb.Th.Tb.N (L5) ↓ Tb.Sp (L5) |
| United States  [Li 2016] | C57BL/6J mice (F; 10 weeks) | *L. rhamnosus* GG (LGG)  VSL#3 (*B. breve, B. longum, B. infantis, L. acidophilus, L. plantarum, L. paracasei, L. bulgaricus, S. thermophilus*) | 1 x 10^9^ CFU/mL *(two times per week)* | acclimatization period ND  4 weeks intervention | OVX (10) OVX+LGG (10) OVX+VSL#3 (10) | LGG: ↑ BV/TV (femur) - BV/TV (spine) -Ct.Ar (femur)  VSL#3: ↑ BV/TV (femur) - BV/TV (spine) - Ct.Ar (femur) |
| United States [Madel 2023] | C57BL/6J mice (F; 6 weeks) | *Saccharomyces boulardii* | 3 g/kg bodyweight CFU ND *(three times per week)* | 2 weeks acclimatization   2 weeks recovery   6 weeks intervention | OVX (9)  OVX+Sb (12) | ↑ BV/TV, Tb.N (femur) ↓ Tb.Sp (femur) - Tb.Th (femur) |
| China  [Yu 2021] | C57BL/6J mice (F; 8 weeks) | *L. plantarum* AR237  *L. plantarum* AR495 | 300 uL of 1 x 10^9^ CFU/mL *(daily)* | 1 week acclimatization   1 week recovery   7 weeks intervention | OVX (8)  AR237 (8)  AR495 (8) | AR237:  - vBMD (femur) ↑ BV/TV, Tb.N, Tb.Th - Tb.Sp (femur)  AR495: ↑ vBMD, BV/TV, Tb.N, Tb.Th (femur) ↓ Tb.Sp (femur) |
| China [Yu 2022] | C57BL/6J mice (F; 8 weeks) | *L. brevis* AR281 | 1 x 10^9^ CFU/mL *(daily)* | 1 week acclimatization   1 week recovery   7 weeks intervention | OVX (8)  OVX+AR281 (8) | ↑ vBMD, BV/TV, Tb.Th, Tb.N (femur) ↓ Tb.Sp (femur) |
| China  [Zhang 2023] | C57BL/6J mice (F; 8 weeks) | *Prevotella histicola* (Ph) | 200 uL of 1 x 10^8^ CFU/mL *(daily)* | 1 week acclimatization   1 week recovery   8 weeks intervention | OVX (6)  OVX+Ph (6) | ↑ vBMD, Tb.N (femur) - BV/TV, Tb.Sp, Tb.Th (femur) |
| China [Wang 2021] | C57BL/6J mice (F; 10 weeks) | *Prevotella histicola* DSM 19854 (Ph) | 0.1mL of 1 x 10^9^ CFU *(every other day)* | acclimatization period ND  12 weeks intervention | OVX (10)  OVX+Ph (10) | ↑ BV/TV, Tb.N (femur) ↓ Tb.Sp, Oc.S/BS, N.Oc/BS (femur) - Tb.Th, Ct.Ar, Ct.Th (femur) |
| China  [Li 2023] | C57BL/6J mice (F; 8 weeks) | *Rothia* | 200 uL of 1 x 10^8^ CFU/mL *(daily)* | 1 week acclimatization   1 week recovery   8 weeks intervention | OVX (6)  ROTHIA (6) | ↑ vBMD, BV/TV, Tb.N, Tb.Th (femur) - Tb.Sp (femur) |
| China [Chen 2023] | C57BL/6J mice (F; 6 weeks) | *L. reuteri* GDMCC1.614 (LR)  *L. acidophilus* GDMCC1.412(LA) | 1 x 10^8-9^ CFU/mL *(daily)* | 2 weeks acclimatization  2 weeks surgery  6 weeks intervention | OVX (10)  OVX+LR (10)  OVX+LA (10)  OVX+LR+LA (10) | LR: - Tb.N, Tb.Th, Tb.Sp, Ct.Th (femur)  LA: ↑ Tb.N, Tb.Th (femur) ↓ Tb.Sp (femur) - Ct.Th (femur)  LR+LA: - Tb.N, Tb.Th, Tb.Sp, Ct.Th (femur) |
| India [Sapra 2022] | C57BL/6J mice (F; 8 weeks) | *B. longum* UBBL-64 M1395 (BL) | 400 uL of 1 x 10^9^ CFU/mL *(daily)* | acclimatization period ND   1 week recovery   7 weeks intervention | OVX (6)  OVX+BL (6) | ↑ vBMD, BV/TV, Tb.Th (femur, tibia, L5); Ct.Th (femur, tibia)  ↓ Tb.Sp (femur, tibia, L5) |
| Switzerland [Wallimann 2021] | Balb/c mice (F; 10 weeks) | *B. longum* 35624® (BBL) | 1 x 10^9^ CFU *(five times per week)* | 2 weeks acclimatization  4 weeks intervention  4 weeks washout period | OVX (7) OVX+BBL (7) | ↑ vBMD (tibia) - BV/TV (tibia) |
| China [Yuan 2022] | Specific pathogen-free mice (F; 6 weeks) | *L. salivarius* LI01 | 3 x 10^9^ CFU/mL *(daily)* | acclimatization period ND  6 weeks intervention | OVX (6)  LI01 (6) | ↑ BV/TV, Tb.N (femur) |
| Korea [Yeom 2021] | Sprague-Dawley rats (F; 9 weeks) | *P. freudenreichii* MJ2 (live, high dose [HLMJ2]; live, low dose [LLMJ2]; inactive, high dose [HDMJ2]; and inactive, low dose [LDMJ2]) *L. plantarum* (LP) | Low:  1 x 10^7^ CFU/mL  High: 1 x 10^8^ CFU/mL *(daily)* | 1 week acclimatization  17 weeks intervention | OVX (8) OVX+LLMJ2 (8) OVX+HLMJ2 (8) OVX+LP (8) OVX+LP+HLMJ2 (8) OVX+LDMJ2 (8) OVX+HDMJ2 (8) OVX+LP+HDMJ2 (8) | LLMJ2 and HLMJ2 (live): - vBMD (femur) - Tt.Ar, Ct.Th (femur)  LP and LP+HLMJ2 (live): ↑ vBMD (femur) -Tt.Ar, Ct.Th (femur)  LDMJ2, HDMJ2, and LP+HDMJ2 (inactive): ↑ vBMD (femur) ↑ Tt.Ar, Ct.Th (femur) |
| Malaysia [Parvaneh 2015] | Sprague-Dawley rats (F; 10 weeks) | *B. longum* ATCC 15707 (BBL) | 1 mL of 1 x 10^8-9^ CFU/mL *(daily)* | 2 weeks acclimatization  2 weeks surgery recovery  16 weeks intervention | OVX (8) OVX+BBL (8) | ↑ vBMD, Tb.Th (femur) - BMC, BV/TV, Po, Tb.Sp, Tb.N (femur) ↑ ObS/BS, OS/BS, OV/BV (femur) ↓ OcS/BS, ES/BS (femur) |
| Malaysia [Parvaneh 2018] | Sprague-Dawley rats (F; 10 weeks) | *L. helveticus* ATCC 27558 (LBH) | 1 mL of 1 x 10^8-9^ CFU/mL *(daily)* | 2 weeks acclimatization  2 weeks surgery recovery  16 weeks intervention | OVX (8) OVX+LBH (8) | ↑ vBMD (femur) - BV/TV, Po, Tb.Th, Tb.Sp, Tb.N (femur) - ObS/BS, OcS/BS, OS/BS, OV/BV, ES/BS (femur) |
| China  [Jia 2021] | Sprague-Dawley rats (F; 10 weeks) | Lifespace, lsPro (*L. rhamnosus* HN001, *B. lactis* BI-04, *B. animals* HN019, *L. fermentum* SBS-1, *L. reuteri* 1e1, *B. longum* BB536, *B. breve* M16-V, *B. infantis* Bi-26, *L. paracasei* Lpc-37) | 1 x 10^7^ CFU *(daily)* | 1 week acclimatization  7 weeks intervention | OVX (5) OVX+lsPro (5) | ↑ BV/TV, Tb.N (femur) ↓ Tb.Pf, Tb.Sp (femur) |
| New Zealand [Kruger 2009] | Sprague-Dawley rats (F; 24 weeks) | *L. rhamnosus* HN001(LR) | 1 x 10^9^ CFU *(daily)* | 2 weeks acclimatization  13 weeks treatment | OVX (15) OVX+LR (15) | Week 4: ↑ BMC (femur) - aBMD, BMC (spine); aBMD (femur)  Week 12: ↑ aBMD, BMC (femur, spine) |
| Iran [Gholami 2020] | Sprague-Dawley rats (F; 12-14 weeks) | *L. acidophilus* (LBA) *L.casei* (LBC) *B.coagulans* (BCO) *Bifidobacterium*(BB) *L. reuteri* (LBR) | 1 mL of 1 x 10^9^ CFU/mL *(daily)* | 1 week acclimatization  4 weeks intervention | OVX (7)  OVX+LBA (7) OVX+LBC (7)  OVX+BCO (7)  OVX+BB (7) OVX+LBR (7) | ↑ aBMD (global, femur, spine, tibia_BB/LBA); BMC (global, femur, tibia_BB) - area (global, femur, tibia, spine); aBMD, BMC (tibia) |
| Iran [Montazeri-Najafabady 2019] | Sprague-Dawley rats (F; 12-14 weeks) | *L. acidophilus* (LBA) *L.casei* (LBC) *B.coagulans* (BCO) *Bifidobacterium*(BB) *L. reuteri* (LBR) | 1 mL of 1 x 10^9^ CFU/mL *(daily)* | 1 week acclimatization   4 weeks intervention | OVX (7)  OVX+LBA (7) OVX+LBC (7)  OVX+BCO (7)  OVX+BB (7) OVX+LBR (7) | ↑ aBMD, BMC (global_LBA/LBC/LBR, femur, tibia_LBC); area (global, femur_LBA/LBC, tibia_LBC, spine_LBA/LBC/BCO) - aBMD, BMC (spine) |
| China  [Guo 2023] | Sprague-Dawley rats (F; 12 weeks) | *LGG* ATCC7469 | 1 mL of 1 x 10^9^ CFU/mL *(daily)* | 1 week acclimatization   6 weeks recovery   6 weeks intervention | OVX (8)  OVX+LGG (8) | ↑ vBMD, Tb.N, Tb.Th (femur) ↓ Tb.Sp (femur) - BV/TV (femur) |
| Republic of Korea [Lee 2023] | Sprague-Dawley rats (F; 6 weeks) | *L. gasseri* (LGA1) | 1 x 10^9^ CFU *(daily)* | 1 week acclimatization   1 week recovery   8 weeks intervention | OVX (6) OVX+LGA1 (6) | - vBMD, BV/TV, Tb.Th, Tb.N, Tb.Sp (femur) |
| Republic of Korea [Lim 2021] | Sprague-Dawley rats (F; 10 weeks) | *L. interstinalis* KCTC 5052  *L. intestinalis* KCCM11812P (YT2)  VSL#3 (*B. breve, B. longum, B. infantis, L. acidophilus, L. plantarum, L. paracasei, L. bulgaricus, S. thermophilus*) | 1 x 10^9^ CFU *(three times per week)* | 4-6 weeks gut normalization (acclimatization)  18 weeks intervention | OVX (10) OVX+VSL#3 (10)  OVX+L.intestinal type strain (11)  OVX+L. interstinalis YT2 (11) | Weeks 8, 12, and 16: ↑ aBMD (whole body) |
| Taiwan [Tsai 2023] | Sprague-Dawle3y rats (F; 12 weeks) | *L. plantarum* GMNL-662 (LP) | Low: 2.07 x 10^8^ CFU/kg  Medium: 4.13 x 10^8^ CFU/kg  High: 8.27 x 10^8^ CFU/kg *(daily)* | acclimatization period ND  14 weeks intervention | OVX (10)  OVX+LP-L (10)  OVX+LP-M (10)  OVX+LP-H (10) | Low:  ↑ BV/TV (femur, L5); vBMD, Tb.N (L5)  ↓ Tb.Sp (femur, L5) - vBMD, Tb.N (femur)  Medium:  ↑ vBMD, BV/TV (femur, L5); Tb.N (L5) ↓ Tb.Sp (femur, L5) - Tb.N (femur)  High:  ↑ vBMD, BV/TV, Tb.N (femur, L5)  ↓ Tb.Sp (femur, L5) |
| Iran [Gholami 2022] | Sprague-Dawley rats (F; 12-14 weeks) | 1: *L. acidophilus, L. casei, B. longum*  2: *L. acidophilus, L. casei, B. coagulans*  3: *L. acidophilus, L. casei, L. reuteri*  4: *L. acidophilus, B. longum, L. reuteri*  5: *L. acidophilus, B. longum, B. coagulans*  6: *L. casei, B. longum, B. coagulans*  7: *L. casei, L. reuteri, B. longum* | 1 mL 1x10^9^ CFU/ml *(daily)* | 1 week acclimatization  4 weeks intervention | OVX (7)  OVX+1 (7)  OVX+2 (7)  OVX+3 (7)  OVX+4 (7)  OVX+5 (7)  OVX+6 (7)  OVX+7 (7) | ↑ aBMD (whole body – treatment 4, 6, 7; spine – treatment 1, 2, 3, 4, 5, 6, 7; femur – treatment 2, 3, 4, 7; tibia – treatment 3, 4) - aBMD (whole body – treatment 1, 2, 3, 5; femur – treatment 1, 5, 6; tibia – treatment 1, 2, 5, 6, 7) |
| Korea [Jin 2022] | Wistar rats (F; 12 weeks) | *L. plantarum* CJNU 3003 (LP) | 400 uL of 1 x 10^8^ CFU/kg *(daily)* | acclimatization period ND  4 weeks recovery   16 weeks intervention | OVX (7)  OVX+LP (7) | - vBMD, BV/TV, Tb.Th, Tb.N, Tb.Sp (tibia) |
| Poland  [Cegiela 2022] | Wistar rats (F; 13 weeks) | *L. rhamnosus* (LR) | 3 x 10^8^ CFU/kg *(daily)* | 1 week acclimatization   1 week recovery   4 weeks intervention | OVX (12)  OVX+LR (10)  OVX+AZM (10)  OVX+AZM+LR (10) | LR - vBMD, BV/TV, Tb.Sp, Tb.N, Ct.Ar, Ma.Ar, Tt.Ar, Ma.Ar/Tt.Ar (femur)  AZM+LR  ↑ BV/TV, Tb.Sp (femoral epiphysis) - vBMD, Tb.N, Ct.Ar, Ma.Ar, Tt.Ar, Ma.Ar/Tt.Ar (femur) |

ABL, alveolar bone loss; AZM, azithromycin; aBMD, areal bone mineral density; BFR, bone formation rate; BMC, bone mineral content; BV/TV, bone volume fraction; Ct.Ar, cortical area; Ct.Ar/Tt.Ar, cortical area fraction; Ct.Th, cortical thickness; Ct.Po, cortical porosity; ES/BS, eroded surface per bone surface; Ma.Ar, medullary area; ND, not defined; ObS/BS, osteoblast surface per bone surface; OcS/BS, osteoclast surface per bone surface; OS/BS, osteoid surface per bone surface; OV/BS, osteoid volume per bone volume; OVX, ovariectomy; Tb.N, trabecular number; Tb.Pf, trabecular pattern factor; Tb.Sp, trabecular separation; Tb.Th, trabecular thickness; TMD; tissue mineral density; Tt.Ar, total area; vBMD, volumetric bone mineral density. ↑ denotes the bone outcome is greater in the probiotic group compared to the control group; ↓ denotes the bone outcomes is lower in the probiotic group compared to the control group; - denotes there is no difference between the probiotic group and the control group.

Table S5. Other models: Summary of findings for the primary bone outcomes from intervention studies using preclinical models for a variety of different conditions or physiological states: high-fat-diet, diabetes, arthritis, fracture healing, tenofovir disoproxil fumarate-induced bone loss, retinoic-induced secondary osteoporosis, CoCrMo particle-induced osteolysis, ulcerative colitis, dorsal incision surgery, glucocorticoid-induced osteoporosis, depression, chronic mild stress, bone cancer pain, cigarette smoking inhalation, antibiotics, genetic modifications and orchidectomy.

| **Country [ref]** | **Rodent species (sex; age)** | **Study model** | **Probiotic intervention** | **Probiotic dose (frequency)** | **Study duration** | **Intervention groups (n)** | **Bone outcomes (bone site of analysis)** |
| --- | --- | --- | --- | --- | --- | --- | --- |
| United States [Behera 2021] | C57BL/6J mice (F; 8 weeks) | high-fat diet | VSL#3 (*B. breve, B. longum, B. infantis, L. acidophilus, L. plantarum, L. paracasei, L. bulgaricus, S. thermophilus*) | 1 x 10^9^ CFU/mL *(every other day)* | acclimatization period ND  8 weeks intervention | HFD (6) HFD+VSL#3 (6) | ↑ vBMD, BV/TV, Tb.N, Tb.Th (femur) ↓ Tb.Sp (femur) ↑ ObN/BS (femur) |
| Spain [Fernandez-Murga 2020] | C57BL/6J mice (M; 6-8 weeks) | high-fat diet | *B. pseudocatenulatum* CECT 7765 (BP) | 1 x 10^9^ CFU/mL *(daily)* | 1 week acclimatization  14 weeks intervention | HFD (10) HFD+BP (10) | ↑ Tb.N (femur) ↓ Tb.Sp (femur) - vBMD, BV/TV, Tb.Th, Tb.Pf (femur) -TMD (femur) |
| [Song 2023] | SPF BALB/c mice (M; 5 weeks) | high-fat diet | *L. coryniformis subsp. Torquens (T3L)* | 1 x 10^9^ CFU/mL *(daily)* | 1 week acclimatization  10 weeks intervention | HFD (10) T3L (10) | ↑ BV/TV, Tb.Th, Tb.Nfemur) ↓ Tb.Sp (femur) - vBMD (femur) |
| Thailand [Eaimworawuthikul 2019] | Wistar rats (M; 6 weeks)` | high-fat diet | *L. paracasei* HII01 (LP) | 1 x 10^8^ CFU/mL *(daily)* | 1 week acclimatization  12 weeks intervention | HFD (6) HFD+LP (6) | ↑ BV/TV (tibia); Tb.Th (tibia) - Tb.Sp, Tb.N (tibia) ↑ MAR, BFR/BS (tibia) ↓ OcS, active erosion surface (tibia) -ObS (tibia) |
| United States [Zhang 2015] | C57BL/6 mice (M; 14 weeks) | diabetes | *L. reuteri* ATCC PTA 6475 (LBR) | 0.3 mL  of 1 x 10^9^ CFU/mL  *(three times per week)* | 1 week acclimatization  4 weeks intervention | D (7) D+LBR (7) | ↑ vBMD, BMC, BV/TV, Tb.N, Tb.Th (femur) ↓ Tb.Sp (femur) ↑ ObS, MAR (femur) - OcS (femur) |
| Slovakia [Rovensky 2005] | Lewis rats (M; ND) | arthritis | *E. faecium* M74 (EF) | 15 mg/kg of 360 x 10^9^/g CFU *(five times per week)* | acclimatization period ND  7 weeks intervention | AA (10) AA+EF (10) | ↑ aBMD (global) |
| [Yeom 2022] | DBA/1J mice (M; 8 weeks) | arthritis | *P. freudenreichii MJ2 (live or dead)* | Low: 1 x 10^7^ CFU/mL  High: 1 x 10^8^ CFU/mL *(not defined)* | 1 week acclimatization  8 weeks intervention | MODEL (8) LLMJ2 (8)  HLMJ2 (8)  LDMJ2 (8)  HDMJ2 (8) | LLMJ2:  ↑ vBMD, Tb.Th (knee joint) ↓ Tb.Sp (knee joint) - BV/TV, Tb.N (knee joint)  HLMJ2: ↑ Tb.Th (knee joint)  - vBMD, BV/TV, Tb.N, Tb.Sp (knee joint)  LDMJ2:  ↑ vBMD, Tb.Th (knee joint) - BV/TV, Tb.N, Tb.Sp (knee joint)  HDMJ2:  ↑ vBMD, BV/TV, Tb.Th, Tb.N (knee joint) ↓ Tb.Sp (knee joint) |
| China [Chang 2022] | Sprague-Dawley rats (ND; 8 weeks) | arthritis | *Clostridium butyricum* GKB7 (CB) | 100 mg/kg bwt of 5.5 x 10^7^ CFU/g  *(daily)* | acclimatization period ND  6 weeks intervention | ACLT (6) ACLT+CB (8) | ↑ vBMD, BMC, BV/TV, BS/TV, Tb.N, Tb.Th (tibia)  ↓ Tb.Sp (tibia) |
| United Kingdom [Sophocleous 2023] | C57BL/6 mice (M; 8 weeks) | arthritis | *Lacticaseibacillus paracasei* 8700:2 DSM13434*, Lactiplantibacillus plantarum* HEAL9 DSM15,312 and *Lactiplantibacillus plantarum* HEAL19 DSM  12,313 (LMIX) | 1 x 10^9^ CFU/mL *(daily)* | acclimatization period ND  10 weeks intervention (*starting two weeks prior to surgery*) | CON (10) LMIX (11) | ↑ BV/TV, Tb.Th, Tb.Pf (femur) - Tb.N, Tb.Sp (femur) |
| China [Lin 2022] | Sprague-Dawley rats (M; 8 weeks) | arthritis | *L. plantarum* GKD7 | 100 mg/kg bwt of 5 x 10^10^ CFU/kg *(daily)* | acclimatization period ND  6 weeks intervention | ACLT (6) ACLT+GKD7 (8) | ↑ vBMD, BMC, BV/TV, BS/TV, Tb.Th, Tb.N (tibia) ↓ Tb.Sp (tibia) |
| United States  [Liu 2020] | C57BL/6J mice (M; 10 weeks) | fracture healing | *B. adolescentis* ATCC 15703 (BA) | 1 x 10^8^ CFU *(five times per week)* | 2 weeks acclimatization  3 weeks intervention | CON (5) BA (5) | ↑ vBMD days 10 and 18, BV/TV days 10, 18, 22, Tb.N days 18 and 22 (L3) ↓ BV/TV day 14 (femur) - BV/TV days 10, 18 and 22 (femur); TV, BV, Tb.Th (femur, L3) |
| Canada [Wang 2023] | C57BL/6 mice (M; 6 weeks) | fracture healing | VSL#3 (*B. breve, B. longum, B. infantis, L. acidophilus, L. plantarum, L. paracasei, L. bulgaricus, S. thermophilus*) | 1 x 10^9^ CFU *(daily)* | Group 1:  5 weeks intervention (pre-fracture)  Group 2:  4 weeks intervention (post-fracture) | PRE-PBS (24) PRE-VSL#3 (24)  POST-PBS (24) POST-VSL#3 (24) | Group 1:  ↑ vBMD, BV/TV (femur callus)  - BV, TV (femur callus)  Group 2:  - vBMD, BV, TV, BV/TV (femur callus) |
| United States [Roberts 2023] | C57BL/6JN mice (F; 73 weeks) | fracture healing | *B. longum* ATCC 15707 | 1 x 10^8-9^ CFU *(daily)* | 4 weeks acclimatization  6 weeks intervention | CON (10) PRO (10) | Day 14 post fracture:  ↑ BV/TV (femur callus); BMC (spine)  ↓ vBMD (spine); BV, TV (femur callus)  Day 21:  ↑ BV/TV, BV (femur callus)  - vBMD BMC (spine); TV (femur callus)  Day 28 post fracture:  ↑ vBMD, BMC (spine)  Day 35 post fracture: ↑ BMC (spine); Tb.Th at 35 days post fracture (L3)  ↓ BS/BV (L3) - vBMD (spine); BV/TV, Tb.N (L3) |
| China  [Liu 2019] | C57BL/6J mice (M; 6 weeks) | tenofovir disoproxil fumarate-induced bone loss | *L. rhamnosus* GG (LGG) | 5 x 10^8^ CFU *(twice per week)* | 1 week acclimatization  4 weeks intervention | TDF (10) TDF+LGG (10) | ↑ vBMD, BV/TV, BS/BV, Tb.N (femur) ↓ Tb.Sp (femur) ↑ Ct.Ar (femur) - BMC, Tb.Th (femur) ↑ MAR, BFR/BS (femur) ↓OcN/BS, OcS/BS (femur) |
| China  [Liu 2020] | Wistar rats (F; 8 weeks) | retinoic-induced secondary osteoporosis | *L. fermentum* ZS40 (ZS40) | 1 mL of 1 x 10^10^ CFU *(daily)* | 1 week acclimatization  6 weeks intervention | RO (6) RO+ZS40 (6) | ↑ vBMD, BV/TV, Tb.N, Tb.Th (femur) ↓ Tb.Sp (femur) |
| China  [Liu 2020] | Wistar rats (F; 8 weeks) | retinoic-induced secondary osteoporosis | *L. plantarum* HFY15 (HFY15) | 1 mL of 1 x 10^10^ CFU *(daily)* | 1 week acclimatization  6 weeks intervention | RO (10) RO+HFY15 (10) | ↑ vBMD, BV/TV, Tb.N, Tb.Th (femur) ↓ Tb.Sp (femur) |
| China [Wang 2017] | (ND; 8 weeks) | CoCrMo particle-induced osteolysis | *L. casei* ATCC 334 (LCA) | 2 x 10^8^ CFU/mL *(three times per week)* | acclimatization period ND  10 weeks intervention | CoPs (5) CoPs+LCA(5) | ↑ BV/TV (calvaria) ↓ Po, erosion area (calvaria) |
| United States [Collins 2016] | Balb/c mice (F; 11 weeks) | dorsal incision surgery | *L. reuteri* ATCC PTA 6475 (LBR) | 0.3 mL of 1 x 10^9^ CFU/mL gavage +  3.3 x 10^8^ CFU/mL water  *(three times per week)* | 1 week acclimatization  8 weeks intervention | DSI (7) DSI+LBR (7) | ↑ BV/TV (femur) - Tb.Th, Tb.N, Tb.Sp (femur) - TMD, BMC, Ec.Pm, Ps.Pm, Ma.Ar, Ct.Ar, Tt.Ar, MOI (femur) - BFR, MAR (femur) |
| United States [Schepper 2020] | C57BL/6J mice (M; 15 weeks) | glucocorticoid-induced osteoporosis | *L. reuteri* ATCC PTA 6475 (LBR) *L. rhamnosus* GG (LGG) | 3.3 × 10^8^ CFU/mL *(daily)* | 1 week acclimatization  8 weeks intervention | GC (8) GC+LBR (8) GC+LGG (8) | LR: ↑ BV/TV (femur, vertebrae); Tb.Th (femur) ↓ Tb.N (femur) - Tb.Sp (femur)  LGG: - BV/TV (femur, vertebrae); Tb.Sp, Tb.Th, Tb.N (femur) |
| [Chargo 2023] | Mice (M; 7 weeks) | glucocorticoid-induced osteoporosis | *L. reuteri* ATCC 6475  VSL#3 (*B. breve, B. longum, B. infantis, L. acidophilus, L. plantarum, L. paracasei, L. bulgaricus, S. thermophilus*) | 300 uL of 1 x 10^9^ CFU/mL *(three times per week)* | 1 week acclimatization  4 weeks intervention | GC (8) GC+LR6475 (7) GC+VSL#3 (8) | LR6475:  ↑ vBMD, BMC, BV/TV, Tb.N (femur, vertebra); Tb.Th (vertebra)  ↓ Tb.Sp (femur, vertebra)  - Tb.Th,TMD, Ct.Th, Ct.Ar, Ma.Ar, Tt.Ar, Ec.Pm, Ps,Pm, MOI (femur)  VSL#3:  - vBMD, BMC, BV/TV, Tb.Th, Tb.N, Tb.Sp (femur, vertebra); TMD, Ct.Th, Ct.Ar, Ma.Ar, Tt.Ar, Ec.Pm, Ps,Pm, MOI (femur) |
| [Jiang 2023] | Rats (M; 8 weeks) | glucocorticoid-induced osteoporosis | *L. plantarum* LP45 | Low: 5 x 10^8^ CFU  Medium: 1 x 10^9^ CFU  High: 2 x 10^9^ CFU *(daily)* | acclimatization period ND  8 weeks intervention | GIO (8) LP45-L (8)  LP45-M (8)  LP45-H (8) | Low:  - aBMD, BMC, BV/TV, Tb.Th, Tb.Sp, Tb.N, Ob.S/BS, Oc.S/BS (tibia, femur)  Medium: ↑ BMC, BV/TV, Tb.Th, Ob.S/BS, (tibia, femur); Tb.N (femur) ↓ Tb.Sp, Oc.S/BS (tibia)  - aBMD, Tb.N (tibia); Tb.Sp, Oc.S/BS (femur)  High:  ↑ aBMD, BMC, BV/TV, Tb.Th, Tb.N, Ob.S/BS (tibia, femur) ↓ Tb.Sp, Oc.S/BS (tibia, femur) |
| China [Li 2023] | Sprague-Dawley rats (F; 12 weeks) | glucocorticoid-induced osteoporosis | *L. plantarum* KRHPS1 (LP) | 1 mL of 1 x 10^9^ CFU/mL *(daily)* | 1 week acclimatization  4 weeks intervention | DEX (5) DEX+LP (5) | ↑ aBMD, BV/TV, Tb.N (femur)  ↓ Tb.Sp (femur)  - Tb.Th (femur) |
| China [Yuan 2023] | Sprague-Dawley (F; 6 weeks) | bone cancer pain | LGG ATCC 53103 | 1mL of 1 x 10^9^ CFU *(daily)* | 1 week acclimatization  1 week intervention | TCI (6)  TCI+LGG (6) | ↑ BV/TV (tibia) |
| China [Xu 2022] | C57BL/6J (M; 6 weeks) | chronic ulcerative colitis | *L. plantarum* GMNL-662 (LP662)  *L. plantarum* 299v (LP299v) | 1 x 10^9^ CFU *(not defined)* | 1 week acclimatization  3 weeks intervention | MODEL (8) LP662 (8)  LP299v (8) | ↑ Tb.Th (femur)  - BV/TV, Tb.N (femur) |
| China [Lan 2022] | C57BL/6J mice (M; 6 weeks) | chronic ulcerative colitis | *B. lactis* BL-99 | Low: 1 x 10^7^ CFU  M: 1 x 10^9^ CFU H: 1 x 10^11^ CFU  *(daily)* | 1 week acclimatization  2 weeks intervention | MODEL (8) LOW (8) MED (8) HIGH (8) | Low:  ↑ Tb.Th (femur)  - BV/TV, Tb.N (femur)  Medium:  - BV/TV, Tb.Th, Tb.N (femur)  High:  ↑ Tb.Th (femur)  - BV/TV, Tb.N (femur) |
| China  [Hao 2019] | Sprague-Dawley rats (M; ND) | depression | *F. prausnitzii* (FP) | 0.2 mL of 1 x 10^9^ CFU *(daily)* | 2 weeks acclimatization  4 weeks CUMS (+intervention)  1 week convalescence  4 weeks recovery (+intervention) | CMS (8) CMS+FP (8) | CMS+FP: - aBMD (global, femur, tibia) |
| South Korea [Lee 2023] | Wistar rats (M; 4 weeks) | chronic mild stress | *B. longum*, *L. helveticus*, and *L. plantarum* (live probiotic, LP; or dead probiotic, DP) | 1 mL of 6 x 10^9^ CFU *(daily)* | 1 week acclimatization  12 weeks treatment | N3 (8)  LPN3 (8)  DPN3 (8) | CMS:  ↑ aBMD, BMC (femur, tibia) |
| Brazil [Tribst 2019] | Wistar rats (M; 3 weeks) | cigarette smoking inhalation | Mixture, MIX (*L. acisophilus, E. faecium, B. subtilis,* and*B. bifidum*) | 2 x 10^9^ CFU/kg *(daily)* | 1 week acclimatization  26 weeks intervention | CSI (8) CSI+MIX (8) | ↑ area, Tb.Th proximal (femur) - aBMD, BMC, Tb.Th medial and distal (femur) |
| United States [Schepper 2019] | BAlb/c mice (M; 11 weeks) | antibiotics | *L. reuteri* 6475 (LBR) *L. rhamnosus* GG (LGG) | 0.3 mL of 1 x 10^9^ CFU/mL *(three times per week)* | 1 week acclimatization  2 weeks antibiotic treatment  4 weeks intervention | ABX (11) ABX+LBR (16) ABX+LGG (9) | LBR: ↑ BV/TV (femur, vertebrae); Tb.N (femur) -Tb.Th, Tb.Sp (femur) ↓ OcS/BS, Oc.N/BS (femur) ↑ BFR (femur) -MAR (femur)  LGG: -BV/TV (femur, vertebrae); Tb.N, Tb.Th, Tb.Sp (femur) - OcS/BS, Oc.N/BS, MAR, BFR (femur) |
| United States  [Tyagi 2018] | C57BL/6 mice (F; 10 weeks) | genetic modification | *L. rhamnosus* GG (LGG) | 1 x 10^9^ CFU *(five times per week)* | acclimatization period ND  4 weeks intervention | CD25 (10)    CD25 LGG (10) DREG (6) DREG LGG (6) DREG-DT (6) DREG-DT LGG (6)  WTLM+DT (6)   WTLM+DT LGG (6) GF (5) GF LGG (5) TCRb (5) TCRb LGG (5) Wnt10b (5) Wnt10b LGG (5)  Wnt10b-/- (5) Wnt10b-/- LGG (5) | CD25 LGG: - BV/TV (spine, femur)  - MAR, BFR/BS, OcN/BS, OcS/BS (femur)  DREG LGG: ↑ BV/TV (spine, femur) ↑ MAR, BFR/BS, OcN  - OcN/BS, OcS/BS (femur)  DREG-DT LGG: - BV/TV (spine, femur) ↑ OcN/BS (femur) - MAR, BFR/BS, OcS/BS (femur)  WTLM+DT LGG: ↑ BV/TV (spine, femur) ↑ MAR, OcS/BS (femur) - BFR/BS, OcN/BS (femur)  GF LGG:  - BV/TV (femur)  TCRb LGG: - BV/TV (spine, femur)  Wnt10b LGG: ↑ BV/TV (spine, femur)  Wnt10b-/- LGG: - BV/TV (spine, femur) |
| Sweden [Lawenius 2022] | C57BL/6J mice (M; 10 weeks) | Orchidectomy | *L. paracasei* DSM 13434*, L. plantarum* DSM 15312 and DSM 15313 (LMIX) | 1 x 10^9^ CFU/mL *(daily)* | acclimatization period ND  6 weeks intervention (starting 2 weeks before surgery) | ORX (13) ORX+LMIX (14) | ↑ vBMD (femur); BV/TV, Tb.N (L5) - aBMD (whole body); Ct. Ar (femur); Tb.Sp, Tb.Th (L5) |

AA, arthritis; aBMD, areal bone mineral density; ABX, antibiotics; BFR/BS, bone formation rate per bone surface; BMC, bone mineral content; BV/TV, bone volume fraction; CAL, clinical attachment loss; CON, control; CoPs, CoCrMo particle-induced osteolysis; CSI, cigarette smoking inhalation; Ct.Ar, cortical area; Ct.Ar/Tt.Ar, cortical area fraction; Ct.Th, cortical thickness; CMS, chronic mild stress; D, diabetes; DSI, dorsal incision surgery; GC, glucocorticoid-induced osteoporosis; GF, germ free; HFD, high-fat diet; Ma.Ar, medullary area; MAR, mineral apposition rate; MOI, cross sectional moment of inertia; ND, not defined; ObN/BPm, number of osteoblasts per bone perimeter; OcN, number of osteoclasts; OcN/BS, number of osteoclast per bone surface; OTM, orthodontic tooth movement; ORX, orchidectomy; Po, total porosity; RA, rheumatoid arthritis; RO, retinoic-induced secondary osteoporosis; Tb.N, trabecular number; Tb.Sp, trabecular separation; Tb.Th, trabecular thickness; TDF, tenofovir disoproxil fumarate-induced bone loss; TMD, tissue mineral density; Tt.Ar, total area; vBMD, volumetric bone mineral density. ↑ denotes the bone outcome is greater in the probiotic group compared to the control group; ↓ denotes the bone outcomes is lower in the probiotic group compared to the control group; - denotes there is no difference between the probiotic group and the control group.

Table S6. List of bacteria strains examined within the intervention studies included in this systematic review. Strains were studied either independently or in combination.

| ***Akkermansia*** | ***Lactobacillus*** |
| --- | --- |
| *muciniphila* | *acidophilus (undefined)* |
| ***Bacillus*** | *acidophilus ATCC 4356* |
| *clausii (undefined)* | *acidophilus GDMCC1.412* |
| *coagulans (L. Sporogenes)* | *brevis AR281* |
| *licheniformis DSM 5749* | *bulgaricus (undefined)* |
| *subtilis (undefined)* | *casei (undefined)* |
| ***Bacteroides*** | *casei ATCC 334* |
| *vulgatus ATCC 8482* | *coryniformis subsp. T3L* |
| ***Bifidobacterium*** | *fermentum SBS-1* |
| *adolescentis ATCC 15703* | *fermentum ZS40* |
| *animalis HN019* | *helveticus (undefined)* |
| *bifidum (undefined)* | *helveticus ATCC 27558* |
| *breve (undefined)* | *interstinalis type strain KCTC 5052* |
| *breve M16-V* | *intestinalis YT2 KCCM11812P* |
| *infantis (undefined)* | *paracasei (undefined)* |
| *infantis BI-26* | *paracasei DSM 13434* |
| *lactis BI-04* | *paracasei GKS6* |
| *lactis BL-99* | *paracasei GMNL-653 (heat-killed)* |
| *longum (undefined)* | *paracasei GMNL-678* |
| *longum 35624®* | *paracasei HII01* |
| *longum ATCC 15707* | *paracasei LMT18-32* |
| *longum BB536* | *paracasei Lpc-37* |
| *longum UBBL-64 (M11395)* | *plantarum (undefined; live or heat-killed)* |
| *pseudocatenulatum CECT 7765* | *plantarum 299v* |
| *thermophilum (undefined)* | *plantarum AR237* |
| ***Clostridium*** | *plantarum AR495* |
| *butyricum GKB7* | *plantarum CJNU3003* |
| ***Enterococcus*** | *plantarum DSM 12313* |
| *faecium (undefined)* | *plantarum DSM 15312 (Lactiplantibacillus)* |
| *faecium M74* | *plantarum DSM 15313 (Lactiplantibacillus)* |
| ***Faecalibacterium*** | *plantarum GKD7* |
| *prausnitzii* | *plantarum GKM3* |
| ***Propionibacterium*** | *plantarum GMNL-662* |
| *freudenreichii MJ2 (live or heat-killed)* | *plantarum HFY15* |
| ***Prevotella*** | *plantarum KACC 15357* |
| *histicola (undefined)* | *plantarum LP45* |
| *histicola DSM 19854* | *plantarum KRHPS1* |
| ***Saccharomyces*** | *reuteri (undefined)* |
| *boulardii* | *reuteri 1e1* |
| ***Streptococcus*** | *reuteri ATCC PTA 6475* |
| *thermophilus* | *reuteri GDMCC1.614* |
|  | *rhamnosus HN001* |
|  | *rhamnosus GG (undefined)* |
|  | *rhamnosus GG ATCC 531.3* |
|  | *rhamnosus GG ATCC 7469* |
|  | *rhamnosus (undefined)* |
|  | *rhamnosus UBLR-58 (MTCC 5402)* |
|  | *salivarius LI01* |

Table S7. Results from the Animal Research: Reporting of In Vivo Experiments (ARRIVE) 2.0 - Essential 10 and Recommended Set (15).

|  | **YES** | **NO** |
| --- | --- | --- |
| **ARRIVE - ESSENTIAL 10** | | |
| Study design - groups compared (a) | 100.00% | 0.00% |
| Study design - experimental unit (b) | 35.21% | 64.79% |
| Sample size - total and group number (a) | 77.46% | 22.54% |
| Sample size - calculation (b) | 11.27% | 88.73% |
| Inclusion and exclusion criteria - a priori (a) | 7.04% | 92.96% |
| Inclusion and exclusion criteria - exclusions (b) | 16.90% | 83.10% |
| Inclusion and exclusion criteria - group sample size (c) | 63.38% | 36.62% |
| Randomisation - methods (a) | 16.90% | 83.10% |
| Randomisation - confounders (b) | 30.99% | 69.01% |
| Blinding | 12.68% | 87.32% |
| Outcome measures - all (a) | 70.42% | 29.58% |
| Outcome measures - primary (b) | 5.63% | 94.37% |
| Statistical methods (a) | 98.59% | 1.41% |
| Statistical methods - assumptions (b) | 18.31% | 81.69% |
| Experimental animals - details (a) | 100.00% | 0.00% |
| Experimental animals - further details (a) | 100.00% | 0.00% |
| Experimental procedures - what and how (a) | 94.37% | 5.63% |
| Experimental procedures - when and how often (b) | 100.00% | 0.00% |
| Experimental procedures - acclimatization (c) | 67.61% | 32.39% |
| Experimental procedures - rationale (d) | 100.00% | 0.00% |
| Results - summary (a) | 97.18% | 2.82% |
| **RECOMMENDED SET** | | |
| Abstract | 29.58% | 70.42% |
| Background - rationale (a) | 100.00% | 0.00% |
| Background - species and model (b) | 100.00% | 0.00% |
| Objectives | 100.00% | 0.00% |
| Ethical statement | 88.73% | 11.27% |
| Housing and husbandry | 92.96% | 7.04% |
| Animal care and monitoring - pain and suffering (a) | 61.97% | 38.03% |
| Animal care and monitoring - adverse events (b) | 11.27% | 88.73% |
| Animal care and monitoring - endpoint (c) | 45.07% | 54.93% |
| Interpretation - results (a) | 100.00% | 0.00% |
| Interpretation - limitations (b) | 39.44% | 60.56% |
| Generalisability | 97.18% | 2.82% |
| Protocol registration | 0.00% | 100.00% |
| Data access | 50.70% | 49.30% |
| Declaration - COI (a) | 97.18% | 2.82% |
| Declaration - funding (b) | 92.96% | 7.04% |


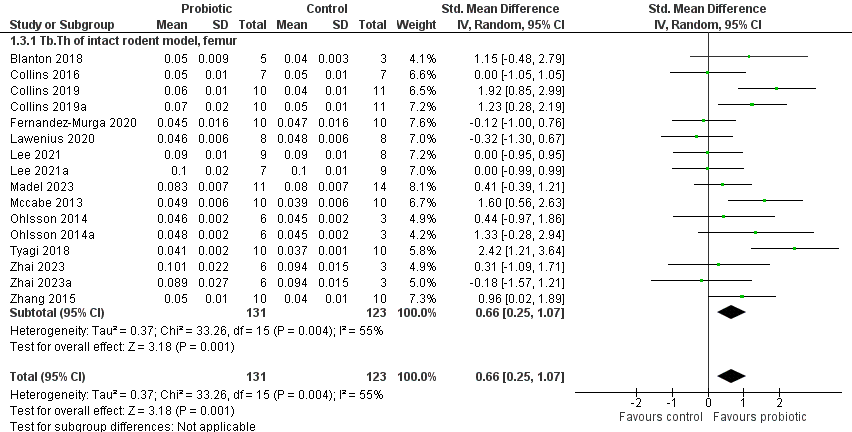


Figure S1. Intact rodent model: The effect of probiotic intervention on trabecular thickness (Tb.Th) of the femur. CI, confidence interval; df, degrees of freedom; IV, weighted mean difference; SD, standard deviation.


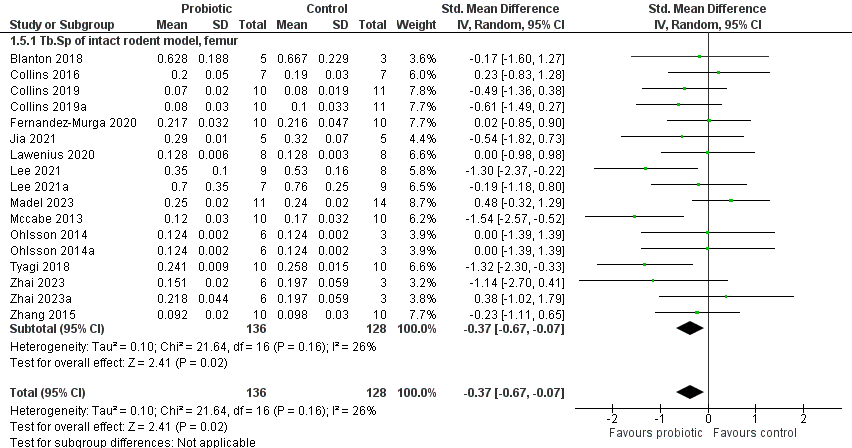


Figure S2. Intact rodent model: The effect of probiotic intervention on trabecular separation (Tb.Sp) of the femur. CI, confidence interval; df, degrees of freedom; IV, weighted mean difference; SD, standard deviation.


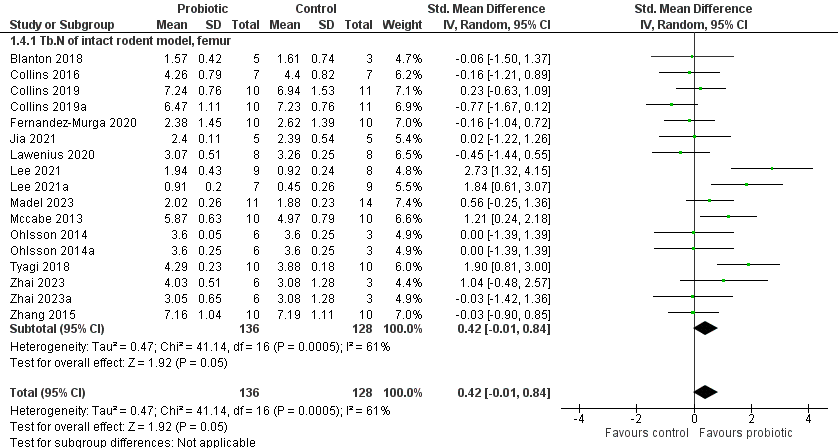


Figure S3. Intact rodent model: The effect of probiotic intervention on trabecular number (Tb.N) of the femur. CI, confidence interval; df, degrees of freedom; IV, weighted mean difference; SD, standard deviation.


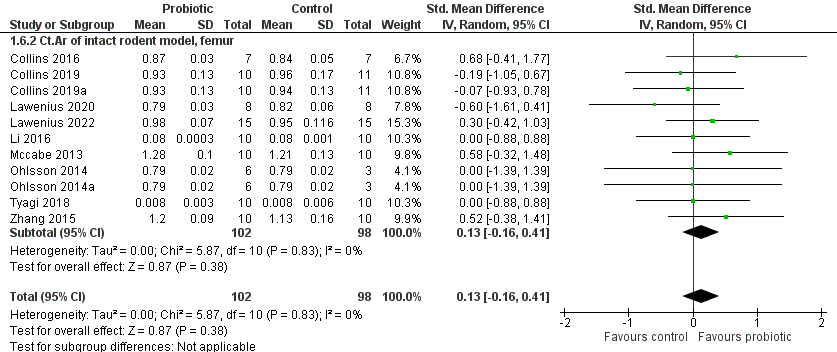


Figure S4. Intact rodent model: The effect of probiotic intervention on cortical area (Ct.Ar) of the femur. CI, confidence interval; df, degrees of freedom; IV, weighted mean difference; SD, standard deviation.

A B C

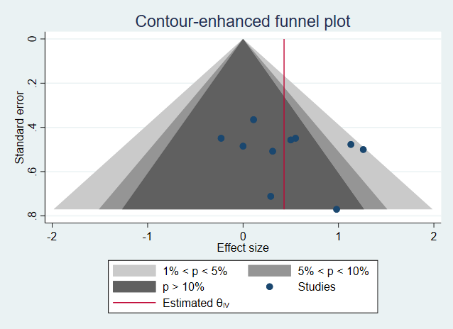

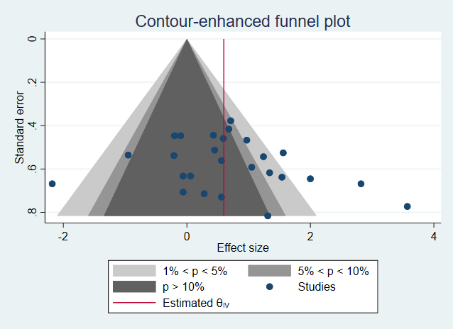

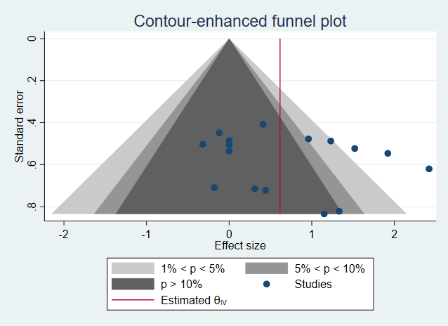

D E F

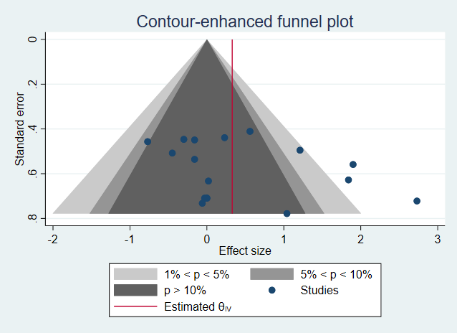

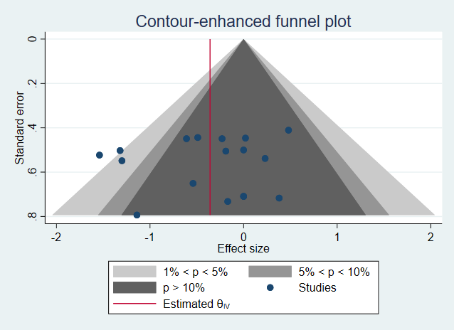

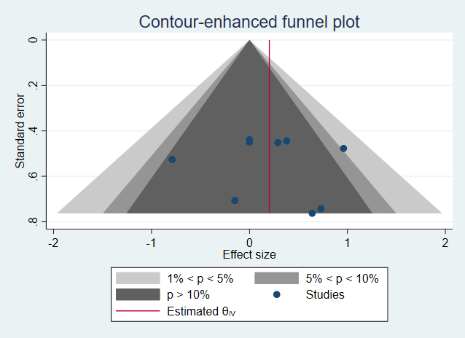

G

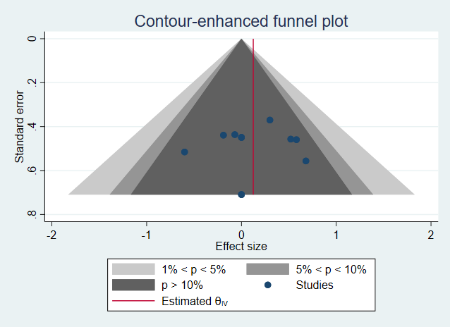


Figure S5. Intact rodent model: Contoured enhanced funnel plots for estimating publication bias of (A) volumetric bone mineral density, vBMD; (B) bone volume fraction, BV/TV; (C) trabecular thickness, Tb.Th; (D) trabecular number, Tb.N (1 study imputed using a trim-and-fill analysis); (E) trabecular separation, Tb.Sp; (F) cortical thickness, Ct.Th; and (G) cortical area, Ct.Ar.


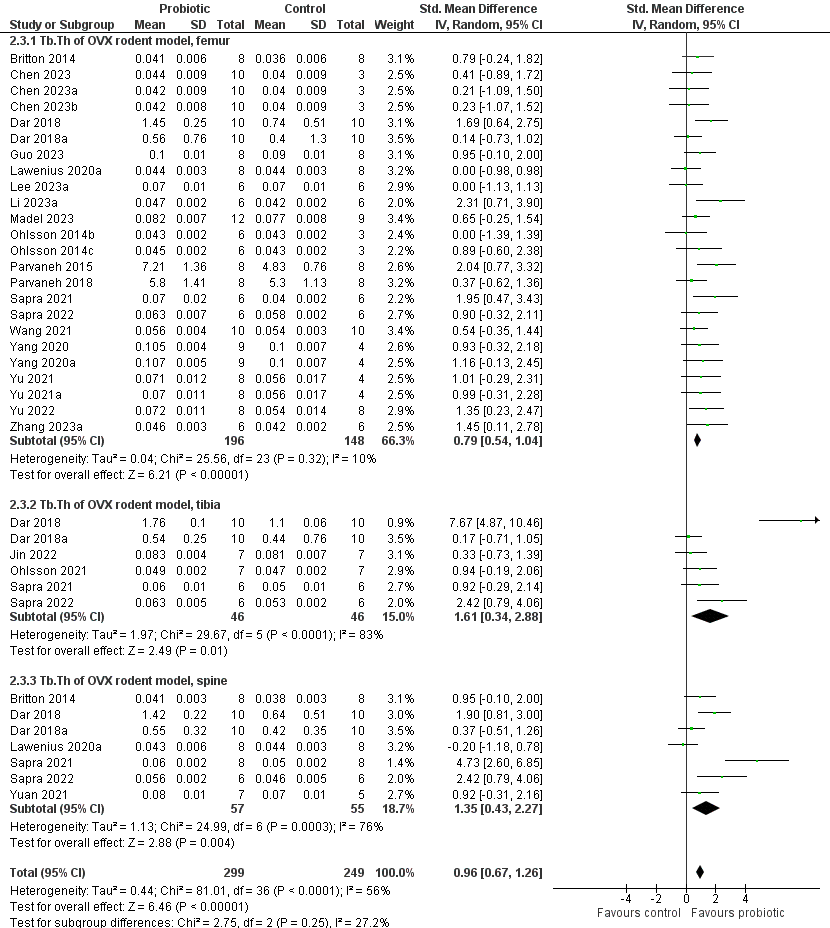


Figure S6. Ovariectomized (OVX) rodent model: The effect of probiotic intervention on trabecular thickness (Tb.Th) of the femur, tibia, and spine. CI, confidence interval; df, degrees of freedom; IV, weighted mean difference; SD, standard deviation.


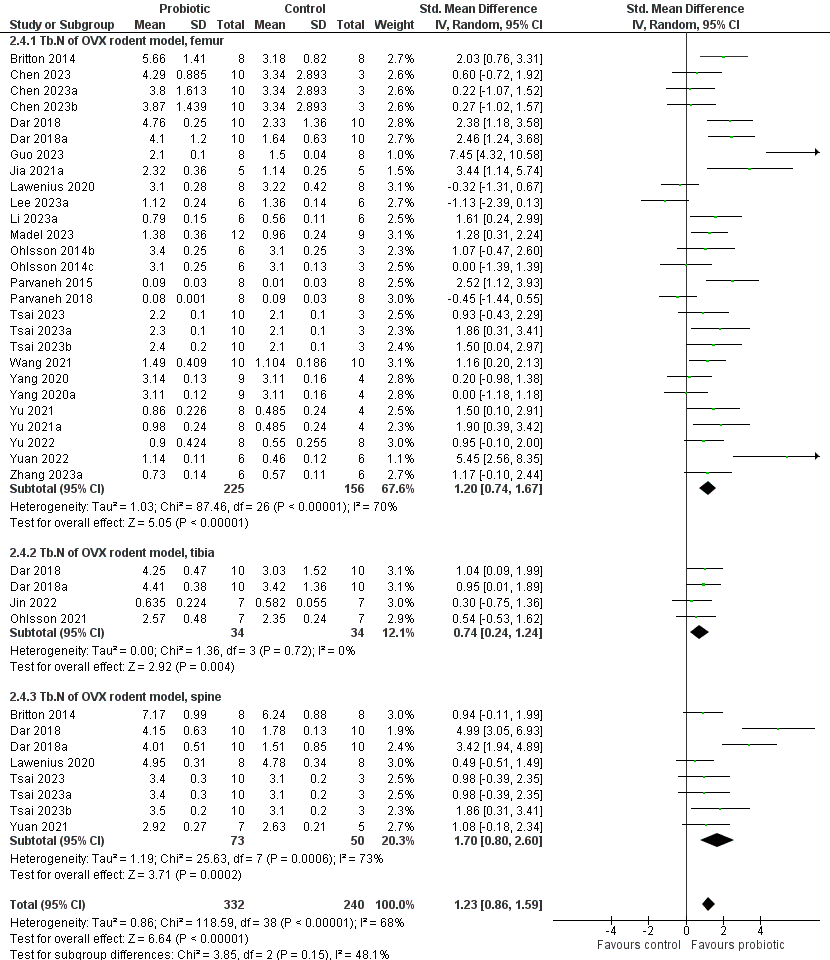


Figure S7. Ovariectomized (OVX) rodent model: The effect of probiotic intervention on trabecular number (Tb.N) of the femur, tibia, and spine. CI, confidence interval; df, degrees of freedom; IV, weighted mean difference; SD, standard deviation.


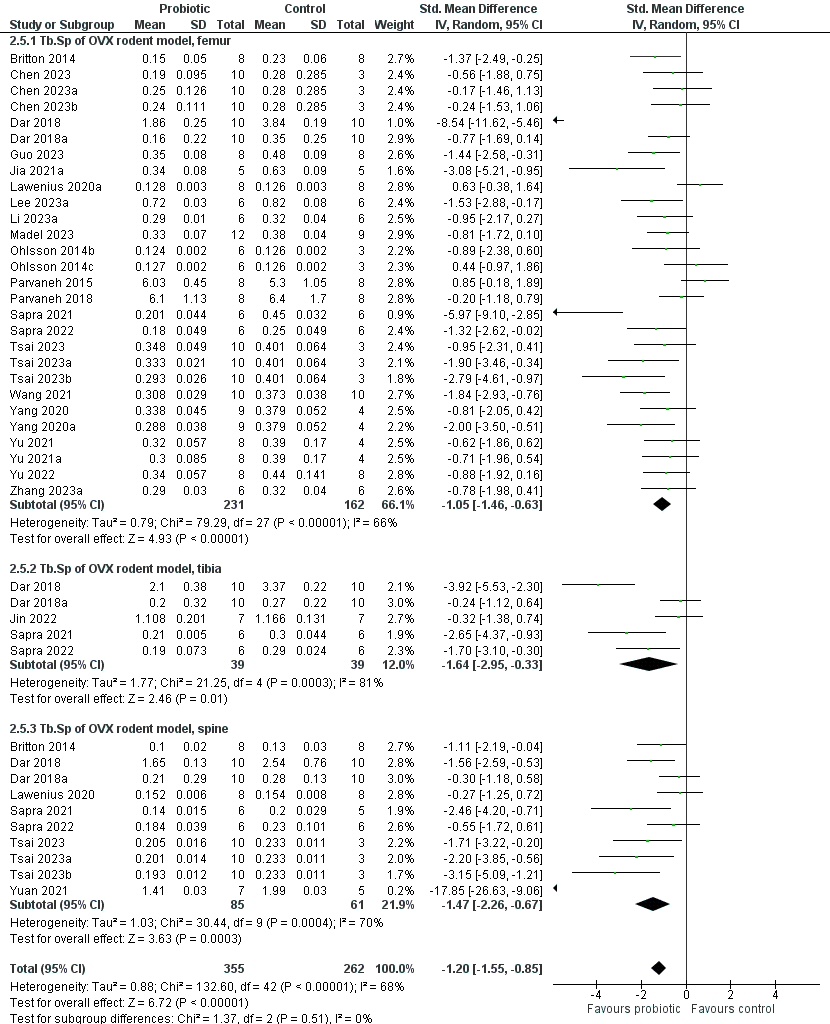


Figure S8. Ovariectomized (OVX) rodent model: The effect of probiotic intervention on trabecular separation (Tb.Sp) of the femur, tibia, and spine. CI, confidence interval; df, degrees of freedom; IV, weighted mean difference; SD, standard deviation.


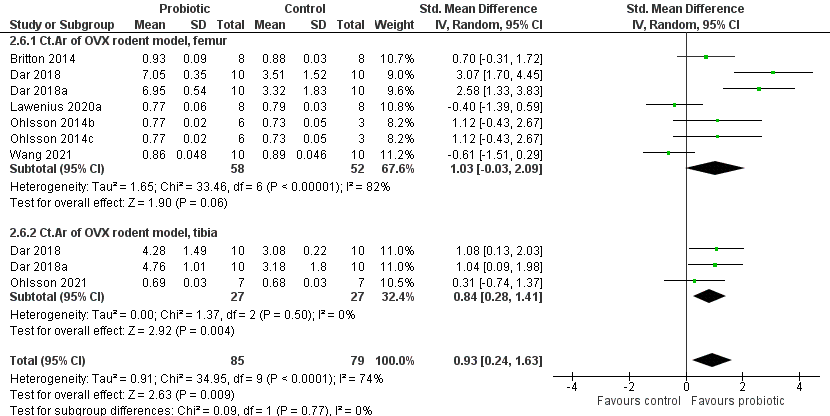


Figure S9. Ovariectomized (OVX) rodent model: The effect of probiotic intervention on cortical area (Ct.Ar) of the femur and tibia. CI, confidence interval; df, degrees of freedom; IV, weighted mean difference; SD, standard deviation.

A B C


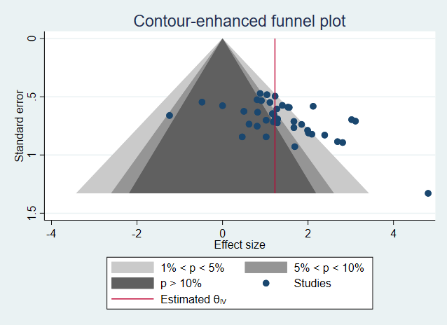

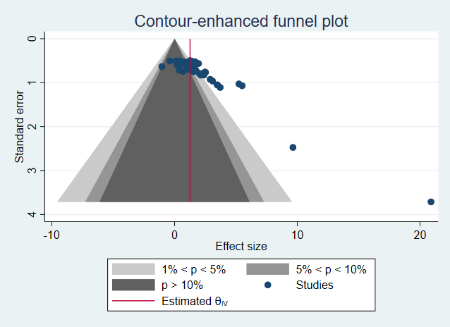

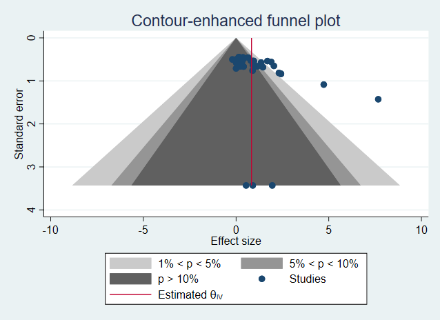

D E F

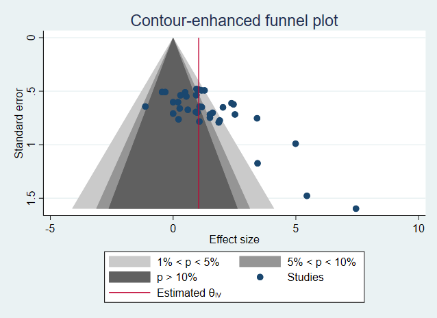

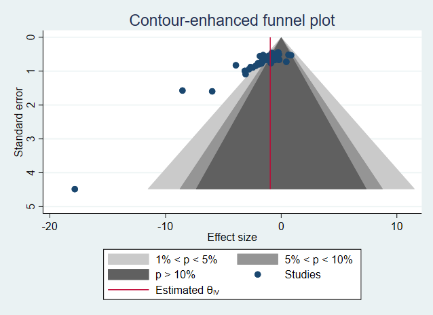

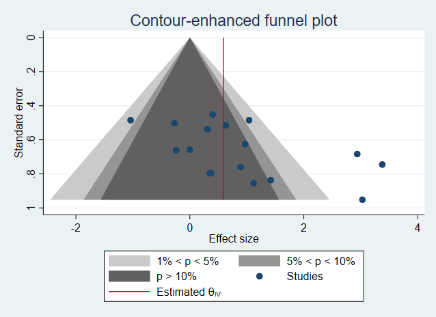

G

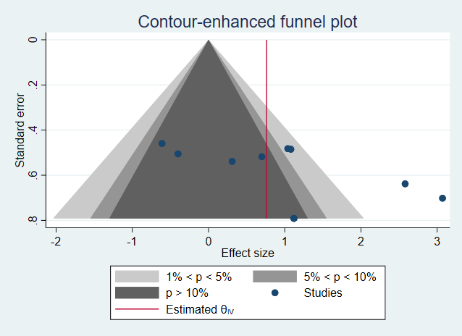


Figure S10. Ovariectomized (OVX) rodent model: Contoured enhanced funnel plots for estimating publication bias for (A) volumetric bone mineral density, vBMD; (B) bone volume fraction, BV/TV; (C) trabecular thickness, Tb.Th; (D) trabecular number, Tb.N; (E) trabecular separation, Tb.Sp (5 studies imputed using a trim-and-fill analysis); (F) cortical thickness, Ct.Th (2 studies imputed using a trim-and-fill analysis); and (G) cortical area, Ct.Ar.
